# *Contactin-associated protein-like 2 (CNTNAP2)* mutations impair the essential α-secretase cleavages, leading to autism-like phenotypes

**DOI:** 10.1038/s41392-024-01768-6

**Published:** 2024-03-01

**Authors:** Qing Zhang, Mengen Xing, Zhengkai Bao, Lu Xu, Yang Bai, Wanqi Chen, Wenhao Pan, Fang Cai, Qunxian Wang, Shipeng Guo, Jing Zhang, Zhe Wang, Yili Wu, Yun Zhang, Jia-Da Li, Weihong Song

**Affiliations:** 1 Oujiang Laboratory (Zhejiang Lab for Regenerative Medicine, Vision and Brain Health), Zhejiang Key Laboratory of Alzheimer’s Disease, Zhejiang Provincial Clinical Research Center for Mental Disorders, School of Mental Health and Wenzhou Kangning Hospital, The Second Affiliated Hospital and Yuying Children’s Hospital, Institute of Aging, Wenzhou Medical University, Wenzhou, Zhejiang 325035 China; 2 Townsend Family Laboratories, Department of Psychiatry, Brain Research Center, The University of British Columbia, 2255 Wesbrook Mall, Vancouver, BC V6T 1Z3 Canada; 3 Chongqing Key Laboratory of Translational Medical Research in Cognitive Development and Learning and Memory Disorders, Ministry of Education Key Laboratory of Child Development and Disorders, Children’s Hospital of Chongqing Medical University, Chongqing, 400014 China; 4 Center for Medical Genetics, Hunan Key Laboratory of Animal Models for Human Diseases, Hunan Key Laboratory of Medical Genetics, Hunan International Scientific and Technological Cooperation Base of Animal Models for Human Diseases, School of Life Sciences, Central South University, Changsha, 410078 Hunan China; 5 The National Clinical Research Center for Geriatric Disease, Xuanwu Hospital, Capital Medical University, Beijing, 100053 China

**Keywords:** Neurodevelopmental disorders, Molecular neuroscience

## Abstract

Mutations in the *Contactin-associated protein-like 2* (*CNTNAP2*) gene are associated with autism spectrum disorder (ASD), and ectodomain shedding of the CNTNAP2 protein plays a role in its function. However, key enzymes involved in the C-terminal cleavage of CNTNAP2 remain largely unknown, and the effect of ASD-associated mutations on this process and its role in ASD pathogenesis remain elusive. In this report we showed that CNTNAP2 undergoes sequential cleavages by furin, ADAM10/17-dependent α-secretase and presenilin-dependent γ-secretase. We identified that the cleavage sites of ADAM10 and ADAM17 in CNTNAP2 locate at its C-terminal residue I79 and L96, and the main α-cleavage product C79 by ADAM10 is required for the subsequent γ-secretase cleavage to generate CNTNAP2 intracellular domain (CICD). ASD-associated *CNTNAP2* mutations impair the α-cleavage to generate C79, and the inhibition leads to ASD-like repetitive and social behavior abnormalities in the *Cntnap2*^*-I1254T*^ knock-in mice. Finally, exogenous expression of C79 improves autism-like phenotypes in the *Cntnap2*^*-I1254T*^ knock-in and *Cntnap2*^*−/−*^ knockout mice. This data demonstrates that the α-secretase is essential for CNTNAP2 processing and its function. Our study indicates that inhibition of the cleavage by pathogenic mutations underlies ASD pathogenesis, and upregulation of its C-terminal fragments could have therapeutical potentials for ASD treatment.

## Introduction

Autism is a common neurodevelopmental disorder characterized by deficits in communication, social interaction, and repetitive/restrictive behaviors and interests. *Contactin-associated protein-like 2* (*CNTNAP2*) gene has been associated with autism, as both rare and common variations have been identified in patients with autism spectrum disorder (ASD).^1,2^ The *Cntnap2* knockout (KO) mouse exhibits autistic phenotypes in communication, social activity, and repetitive behaviors, along with pathological changes in neurodevelopment,^3^ and it has been widely used as an animal model for ASD study.^4–8^ However, the mechanism underlying the role of CNTNAP2 in autism remains elusive.

Human CNTNAP2 is a type-1 transmembrane protein of 1331 amino acids (aa) with three lobes.^9–11^ The gene encoding for the human CNTNAP2 protein locates in chromosome 7, and we reported that intermittent hypoxia treatment enhanced *CNTNAP2* transcription to upregulate its protein expression.^12^ The CNTNAP2 protein belongs to the CNTNAP subfamily of the Neurexin family and functions as a cell adhesion molecule.^11,13^ It was initially found in a complex with voltage-gated potassium channel Kv1 and transient axonal glycoprotein-1 (TAG-1) in the juxtaparanodal region.^14,15^ CNTNAP2 is also located at pre- and post-synaptic sites,^16,17^ and it plays an essential function in axonal growth,^18^ dendritic arborization,^19^ and spine development.^17,20^ Our recent study has demonstrated that CNTNAP2 full-length protein and its C-terminal fragments (CTF) have a half-life of approximately 3–4 h. Its degradation is dependent on both ubiquitin-proteasome system (UPS) and the macroautophagy-lysosome pathway, while the latter is the more common way for CNTNAP2 degradation.^21^

Some synaptic molecules, such as neurexin 3β and neuroligin-1, undergo ectodomain shedding.^22,23^ Several type-1 transmembrane proteins, like amyloid precursor protein (APP) and Notch, also go through proteolytic processing to achieve their functions.^24–26^ Both APP and Notch can be cleaved directly by α-secretase and then γ-secretase. Three members of the A Disintegrin And Metalloproteinase (ADAM) family have been identified as candidates for the α-secretase: ADAM10, ADAM17 and ADAM9. While ADAM10 is widely expressed in the central nervous system, ADAM17 is more restrictedly expressed in the hippocampus. Both ADAM10 and ADAM17 are cell membrane-bound proteases that cleave substrates including growth factors, receptors and cytokines primarily at the extracellular sites close to the cell membrane. APP is a single-pass transmembrane protein with a large extracellular N-terminal domain, and a shorter cytoplasmic C-terminal tail. Under physiological conditions, APP is predominantly cleaved by α-secretase between Lys-16 and Leu-17 within the amyloid β protein (Aβ) sequence to create sAPPα and CTFα C83, and the resulting C83 is subsequently cleaved by the γ-secretase to yield P3α and CTFγ.^27^ In the non-amyloidogenic pathway, ADAM10 is the constitutive α-secretase, while ADAM17 functions in the regulated α-secretase cleavage of APP. In the Golgi, Notch is cleaved by furin (S1 cleavage) and glycosylated by O-fucosyltransferase. The processed Notch receptor is then transported to the cell membrane, where it binds to the Delta ligand. Upon ligand binding, the Notch extracellular domain is cleaved away by ADAM10/ ADAM17- dependent α-secretase (S2 cleavage). The membrane-bound fragment is further cleaved by γ-secretase (S3 cleavage) to release the Notch intracellular domain (NICD). The proteolytic processing of Notch to generate NICD is critical for normal development.^25^ γ-secretase is an enzyme complex that contains presenilins (PS) as its core component. This high molecular weight complex also requires the presence of nicastrin, anterior pharynx-defective 1 (APH-1), and PEN-2 for its enzymatic activity.^28^ PS1 and PS2 are homologous with 67% identical sequences. They are both ubiquitously expressed in human and mouse tissue, while PS1 mRNA levels are significantly higher in developing brains. It has been confirmed that a total elimination of γ-secretase activity in the PS1/PS2 double knockout cells established the central role of presenilins in the γ-secretase complex. PS1-deficient neurons had a markedly reduced APP γ-secretase cleavage and Aβ generation, arguing for PS1 as the principal component of the γ-secretase complex.^29^ PS1 is also required for the γ-cleavage of Notch.^25^

Like APP and Notch, CNTNAP2 has a long extracellular N-terminal and a short intracellular C-terminal. CNTNAP2 is cleaved by matrix metalloproteinase 9 (MMP9), and the shed ectodomain regulates Ca^2+^ homeostasis and neuronal network synchrony.^30^ Recently, we found that CNTNAP2 is cleaved by presenilins-dependent γ-secretase to produce the CNTNAP2 intracellular domain (CICD), and expression of the CICD improves autism-like behaviors.^31^ Our current study reveals that in addition to its γ-secretase cleavage, CNTNAP2 undergoes complex proteolytic processing by furin and a disintegrin and metalloproteinase (ADAM)10/17-dependent α-secretase. The main α-cleavage product C79 by ADAM10 is required for the subsequent γ-secretase cleavage to generate the CICD. Furthermore, ASD-associated pathogenic CNTNAP2 mutations inhibited the α-cleavage, leading to autism-like phenotypes in vivo, whereas exogenous expression of the α-cleavage product C79 improves autism-like phenotypes in the *Cntnap2*^*-I1254T*^ knock-in and *Cntnap2*^*−/−*^ knockout mice.

## Results

### CNTNAP2 is cleaved by furin and α-secretase

**Fig. 1:**
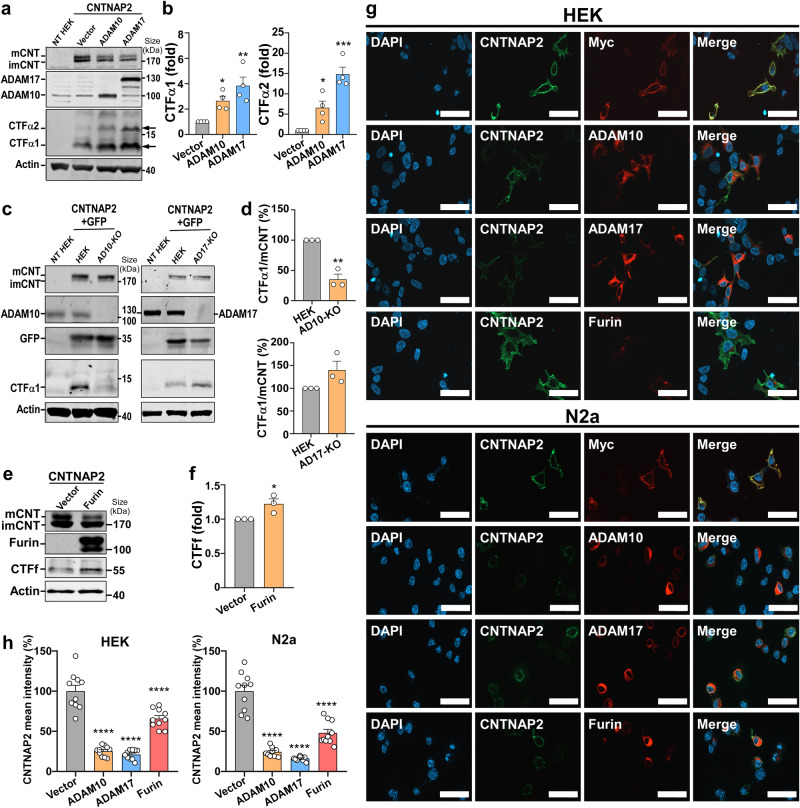
CNTNAP2 is cleaved by α-secretase and furin. **a**, **b** ADAM10 and ADAM17 cleaved mature CNTNAP2 (mCNT) in HEK cells. HEK cells were co-transfected with CNTNAP2 and empty vector, ADAM10 or ADAM17 plasmids, and harvested 24 h after transfection for Western Blot analysis. Non-transfected HEK (NT HEK) was used as a negative control. CTFα1 and CTFα2 were increased/generated by ADAM10 and ADAM17. *n* = 4 independent experiments, ordinary one-way ANOVA followed by Dunnett’s multiple comparisons test, *p < 0.05; **p < 0.01; ***p < 0.001. **c**, **d** ADAM10 is the major α-secretase that generates CTFα1. CNTNAP2 was co-transfected with GFP into HEK, ADAM10-knockout (KO), or ADAM17-KO HEK cells. The CTFα1/mCNT ratio was reduced in ADAM10-KO cells while increased in ADAM17-KO cells. *n* = 3 independent experiments, unpaired *t*-test, **p < 0.01. **e**, **f** Furin cleaved mature CNTNAP2 in HEK cells. HEK cells were co-transfected with CNTNAP2 and vector or furin. CTFf at 55 kDa was increased by furin. *n* = 3 independent experiments, unpaired *t*-test, *p < 0.05. **g** CNTNAP2 on the cell membrane was cleaved in HEK and N2a cells. CNTNAP2 was co-transfected with vector (myc), ADAM10, ADAM17, or furin plasmids into HEK and N2a cells. Cells were fixed 24 h after transfection, and immunocytochemistry (ICC) was performed. CNTNAP2 was detected by an anti-CNTNAP2 antibody targeting the N-terminal 1001–1042 aa outside the cell membrane. CNTNAP2 was mainly expressed on the cell membrane and was reduced by co-transfected plasmids. Images were acquired using Zeiss Apotome to reflect the morphology. Scale bar represents 50 μm. **h** Quantification of ICC in HEK and N2a. CNTNAP2 mean intensity was measured using the corresponding conventional fluorescence images in Supplementary Fig. 2. *n* = 10 cells from 2 independent experiments, ordinary one-way ANOVA followed by Dunnett’s multiple comparisons test, ****p < 0.0001. All the results are expressed as mean ± SEM

To reveal the proteolytic processing pathway of CNTNAP2, we first investigated whether CNTNAP2 can be cleaved by ADAM10/17-dependent α-secretase. pRK5-CNTNAP2 was co-transfected with an empty vector, pRK5M-ADAM10 or pRK5M-ADAM17, into HEK293 cells. Two bands of CNTNAP2 full-length protein were detected at 170 kDa, the higher glycosylated mature form and the lower immature form (Fig. 1a). Mature CNTNAP2 was reduced by ADAM10 to 33. 11 ± 3.97% (*p* < 0.0001) and by ADAM17 to 32.48 ± 2.16% (*p* < 0.0001) compared with the control, while immature CNTNAP2 was not significantly affected (Supplementary Fig. 1a). ADAM10 and ADAM17 cleaved CNTNAP2 to generate two C-terminal fragments (CTFs) at ~15 kDa, a predominant CTFα1 and a weaker CTFα2. Expression of ADAM10 and ADAM17 increased CTFα1 to 2.65 ± 0.36 folds (*p* = 0.0483) and 3.84 ± 0.68 folds (*p* = 0.0026), while markedly elevated CTFα2 to 6.56 ± 1.63 (*p* = 0.0332) folds and 14.83 ± 1.72 folds (*p* < 0.0001), respectively (Fig. 1b). To validate the cleavage of CNTNAP2 by α-secretase, we generated ADAM10-KO and ADAM17-KO HEK cell lines via the CRISPR/Cas9 technique. Genetic ablation of ADAM10 drastically reduced CTFα1, while ablation of ADAM17 reduced the CTFα2 level without significantly affecting CTFα1 production (Supplementary Fig. 1c). While the CTFα1/mCNTNAP2 ratio was reduced to 35.2 ± 8.72% (*p* = 0.0017) in ADAM10-KO cells, the ratio was increased to 139.9 ± 19.54% (*p* = 0.1106) in ADAM17-KO cells (Fig. 1c, d), due to the compensatory effect between ADAM10 and ADAM17. Our data clearly demonstrates that mature CNTNAP2 is cleaved into two CTFα by both ADAM10 and ADAM17: the major α-secretase product CTFα1 is predominantly generated by ADAM10, and the minor α-secretase product CTFα2 is predominantly produced by ADAM17, respectively.

We next investigated whether CNTNAP2, like the Notch-1 protein, could also be cleaved by furin. Furin cleaved mature CNTNAP2 to 52.87 ± 5.86% (*p* = 0.0013) (Supplementary Fig. 1b) and enhanced a CTFf at 55 kDa to 1.22 ± 0.07 folds (*p* = 0.0413) in HEK cells (Fig. 1e, f). The results were further confirmed by furin inhibitor treatment (Fig. 3a–d). Consistently, full-length CNTNAP2 was reduced to 39.42 ± 6.65% (*p* = 0.0028) by ADAM10, 21.13 ± 3.73% (*p* = 0.0005) by ADAM17, and 51.53 ± 15.48% (*p* = 0.0104) by furin, while CTFα2 was increased to 3.04 ± 0.27 and 8.09 ± 1.89 (*p* = 0.002) folds, and CTFf was elevated to 4.87 ± 1.81 (*p* = 0.041) folds by corresponding proteases in mouse neuroblastoma Neuro-2a (N2a) cells (Supplementary Fig. 1d, e). CNTNAP2 is mainly expressed on the cell membrane of both HEK and N2a cells (Fig. 1g). Consistent with Western blot results, overexpression of ADAM10, ADAM17, or furin drastically reduced CNTNAP2 level on the membrane to 25.27 ± 2.02%, 21.06 ± 1.86%, or 65.82 ± 3.74% in HEK cells, and 24.41 ± 1.74%, 16.13 ± 0.84%, or 47.91 ± 4.54% in N2a cells (*p* < 0.0001 in all the comparisons with controls) (Fig. 1h, Supplementary Fig. 2). Collectively, our results demonstrated that CNTNAP2 undergoes proteolytic processing by furin and ADAM10/17-dependent α-secretase.

### Identification of α-cleavage sites on CNTNAP2

**Fig. 2:**
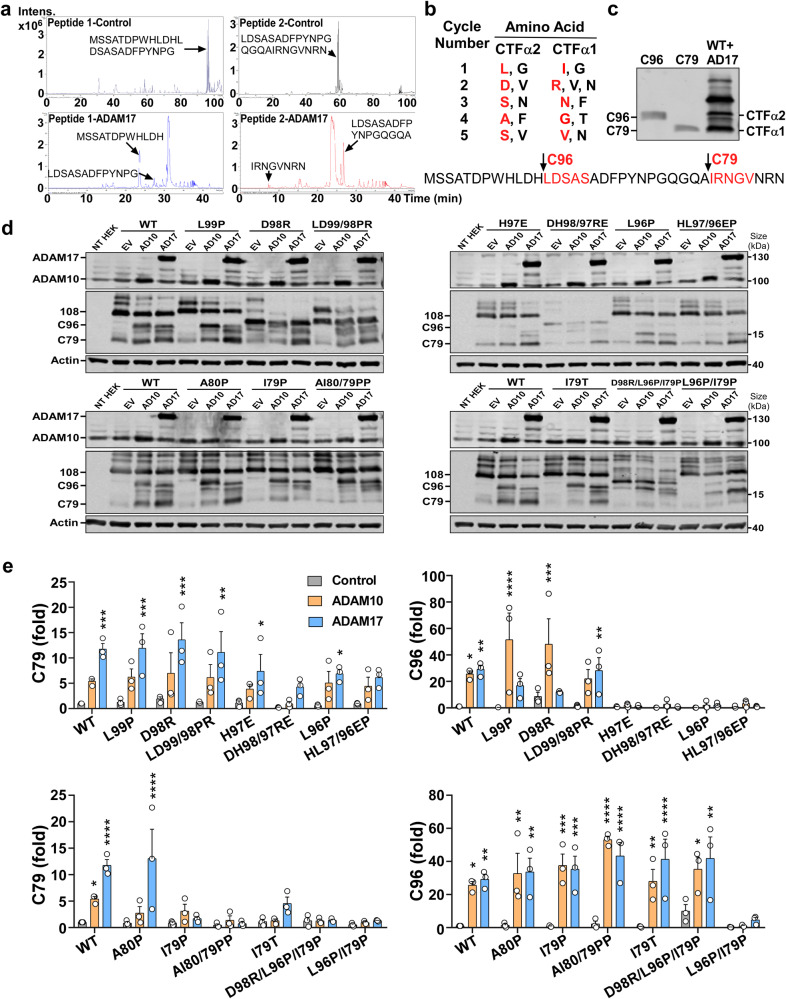
Identification of α-secretase cleavage sites on CNTNAP2. **a** Peptide in vitro cleavage. Two peptides were incubated in the assay buffer with or without ADAM17 at 37 °C for 24 h and then were sent to Mass Spectrometry analysis. The extracted ion chromatogram showed two intact peptides (top) and cleaved complementary fragments (bottom). Peptide 1 (M108-G84) was cleaved by ADAM17 at 97H/L96. Peptide 2 (L96-N72) was cleaved at 80 A/79I. **b** N-terminal sequencing results identified the first 5 amino acids (aa) of CTFα1 as IRNGV and the first 5 aa of CTFα2 as LDSAS. The bottom sequence shows the last 108 to 72 aa of CNTNAP2 from the C-terminus and α-secretase cleavage sites at L96 and I79. **c** Protein ladders of the C-terminal CNTNAP2. C79 corresponded to the size of CTFα1, and C96 showed the same size as CTFα2. **d**, **e** Mutations at the α-secretase cleavage sites L96 and I79 affected CNTNAP2’s cleavage. Wildtype (WT) or mutant plasmids were co-transfected with empty vector (EV), ADAM10 (AD10), or ADAM17 (AD17) into HEK cells. Alterations in the migration rate were observed in constructs containing D98R. Two upper/lower blots show samples from the same experiment. The parallel blots were processed in the same electrophoresis chamber and scanned together simultaneously. *n* = 3 independent experiments, two-way ANOVA followed by Dunnett’s multiple comparisons test (compare ADAM10 and ADAM17 with Control for each plasmid), row factor = mutation, column factor = ADAM10/17 overexpression. *p < 0.05; **p < 0.01; ***p < 0.001; ****p < 0.0001. All the results are expressed as mean ± SEM

To identify the α-secretase cleavage sites, plasmids expressing the last 178, 108, and 91 aa of CNTNAP2 C-terminus were co-transfected with ADAM10 or ADAM17. CTFα1 and CTFα2 were generated from both 178 and 108 constructs while only CTFα1 was produced from the 91 construct, indicating that the α-secretase site generating CTFα2 is within the last 108 aa, and the CTFα1 is within the last 90 aa (Supplementary Fig. 3a). Next, Mass Spectrometry and N-terminal sequencing were performed to identify the cleavage sites. Synthetic peptide 1 (C-terminal 108-84 aa of CNTNAP2) and peptide 2 (C-terminal 96-72 aa) were treated with the recombinant human ADAM17 protein, and Mass Spectrometry results showed that ADAM17 cleaved peptide 1 between H97 and L96 and peptide 2 between A80 and I79 (Fig. 2a). N-terminal sequencing also identified that CTFα1 started from IRNGV, and CTFα2 started from LDSAS (Fig. 2b, Supplementary Fig. 3b). In addition, we generated truncated CNTNAP2 C-terminal protein ladders C79 and C96, which perfectly matched the size of CTFα1 and CTFα2 (Fig. 2c). These results clearly demonstrated that CNTNAP2 is cleaved by α-secretase primarily at I79 to generate C79 (CTFα1), and less at L96 to yield C96 (CTFα2), respectively.

Next, plasmids expressing the 108aa protein containing mutations at α-secretase sites were co-transfected with ADAM10 or ADAM17 into HEK cells. ADAM10 and ADAM17 cleaved the wildtype (WT) construct and markedly enhanced the C96 levels to 25.63 ± 2.21 and 29.19 ± 3.02 folds, respectively (Fig. 2d, e). However, C96 levels were only increased to 1.98 ± 0.86 and 1.41 ± 0.45 folds in H97E, and to 2.07 ± 1.66 and 2.11 ± 1.10 folds in L96P, respectively, by ADAM10 and ADAM17. The C96 generation was also blocked in DH98/97RE and HL97/96EP mutant constructs (Fig. 2d, e). C79 levels in WT were increased by ADAM10 to 5.40 ± 0.46 folds and by ADAM17 to 11.76 ± 1.10 folds, respectively (Fig. 2d, e). However, C79 generation was barely changed to 3.11 ± 1.29 and 1.69 ± 0.39 folds in I79P, and to 1.37 ± 0.84 and 0.76 ± 0.29 folds in AI80/79PP by overexpressed ADAM10 and ADAM17 (Fig. 2d, e). These results demonstrated that H97E and L96P mutations reduced C96 generation, whereas I79P and AI80/79PP blocked C79 generation. Notably, I79T corresponding to an ASD-associated I1253T mutation was found in a family with two autism patients.^2^ I79T significantly inhibited C79 generation, only reaching 1.29 ± 0.32 and 4.55 ± 1.21 folds upon ADAM10/17 overexpression (Fig. 2d, e). These results demonstrated that mutations at the α-cleavage sites L96 and I79 significantly affect CNTNAP2’s cleavage (Supplementary Table 1). In summary, ADAM10/17 cleaves CNTNAP2 at L96 and I79, and the ASD-associated pathogenic mutation I79T inhibits the α-cleavage-mediated C79 generation.

### Sequential cleavage of CNTNAP2 by furin, α- and γ-secretase

**Fig. 3:**
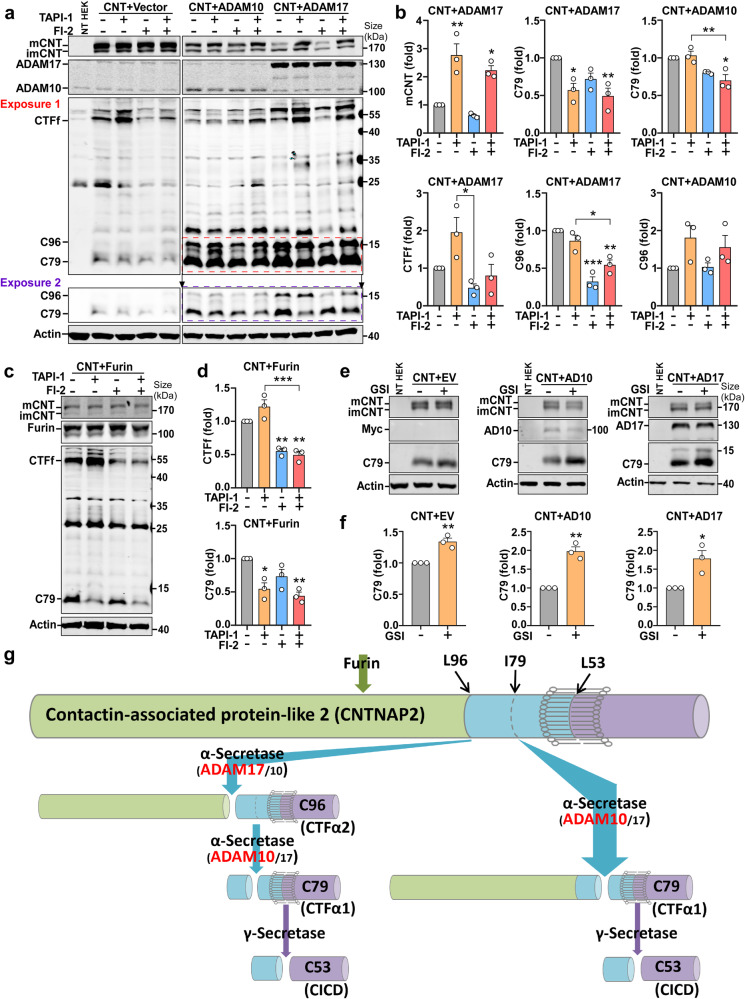
Sequential cleavages of furin, α- and γ-secretase. **a** Western blot of control (Vector), ADAM10 overexpressing, and ADAM17 overexpressing HEK cells. Exposure 1 was used to analyze CTFf in the ADAM17 overexpressing cells and C96 in the ADAM10 overexpressing cells; exposure 2 was used for assessing C79 and C96 in the ADAM17 overexpressing cells. TAPI-1 (10 μM) is an ADAM17-specific inhibitor; FI-2 (20 μM) stands for Furin inhibitor-2. **b** Quantification of the dynamic changes of mature CNTNAP2 and CTF levels in the ADAM10/ADAM17 transfected cells upon inhibitor treatments in (**a**). *n* = 3 independent experiments, ordinary one-way ANOVA followed by Tukey’s multiple comparisons test, *p < 0.05; **p < 0.01; ***p < 0.001. *P* values stand for comparisons with the Control group unless noted by the brackets. **c**, **d** Dynamic changes of CTFs in the furin transfected cells, showing the furin-ADAM17 cleavages. *n* = 3 independent experiments, ordinary one-way ANOVA followed by Tukey’s multiple comparisons test, *p < 0.05; **p < 0.01; ***p < 0.001. *P* values stand for comparisons with the Control group unless noted by the brackets. **e** Western Blot of control (Vector), ADAM10 overexpressing, and ADAM17 overexpressing HEK cells. GSI is γ-secretase inhibitor L-685,458 (20 nM). **f** Quantification of the sequential cleavage by α- and γ-secretase in (**e**). *n* = 3 independent experiments, unpaired *t*-test, *p < 0.05, **p < 0.01. **g** Schematic of CNTNAP2 processing. Mature CNTNAP2 undergoes sequential cleavages by furin and α-, γ-secretase. Furin cleaves CNTNAP2 to generate CTFf, which is further processed by α-secretase. α-secretase ADAM10 and ADAM17 share the same cutting sites but have different site preferences. ADAM10 primarily cleaves at I79 to produce the predominant C79, and ADAM17 preferably cleaves at L96 to produce the weaker C96. C96 is subsequently processed by α-secretase into C79. γ-secretase further cleaves C79 at L53 within the transmembrane domain to generate CICD. All the results are expressed as mean ± SEM

To further examine the processing pathway of CNTNAP2 by furin and α-secretase, ADAM17 overexpressing cells were treated with ADAM17 inhibitor TAPI-1 and furin inhibitor-2 FI-2. TAPI-1 significantly elevated mature CNTNAP2 to 2.77 ± 0.41 folds (*p* = 0.0021) and reduced C79 level to 0.57 ± 0.09 fold (*p* = 0.0215), while it did not affect mature CNTNAP2 or C79 in the ADAM10-transfected cells (Fig. 3a, b). TAPI-1 also increased CTFf to 1.95 ± 0.40 folds, and FI-2 decreased CTFf to 0.47 ± 0.11 fold (*p* = 0.0148 compared with TAPI-1) in the ADAM17-transfected cells, while the combination of TAPI-1 and FI-2 brought the CTFf to 0.80 ± 0.30 fold, a level between TAPI-1 and FI-2 treatment alone (Fig. 3a, b, CNT + ADAM17 graphs), suggesting that furin-generated CTFf is further cleaved by ADAM17. The downstream CTF patterns also support this sequential cleavage. TAPI-1 reduced C79 level to 0.57 ± 0.09 fold (*p* = 0.0215) by inhibiting ADAM17 activity. FI-2 marginally decreased C79 to 0.72 ± 0.08 fold by reducing CTFf for the subsequent ADAM17 cleavage. As expected, TAPI-1 + FI-2 significantly reduced the C79 level to 0.49 ± 0.11 fold (*p* = 0.0086) in an additive manner. While TAPI-1 did not affect the C96 level (0.86 ± 0.08 fold), FI-2 significantly reduced C96 with (0.54 ± 0.07 fold, *p* = 0.0028) or without TAPI-1 (0.32 ± 0.06 fold, *p* = 0.0002) (Fig. 3a, b, CNT + ADAM17 graphs), indicating that C96 was also sequentially cleaved from CTFf by ADAM17. These results demonstrated that mature CNTNAP2 is sequentially cleaved by furin to generate CTFf, and subsequently, CTFf is cleaved by ADAM17 to generate C96 and C79.

Next, we examined the cleavage in ADAM10 overexpressing cells. While TAPI-1 alone did not affect the C79 level (1.03 ± 0.06 folds), TAPI-1 and FI-2 together significantly reduced the C79 level to 0.70 ± 0.08 fold, compared with control (*p* = 0.0103) and TAPI-1 treatment (*p* = 0.0058), suggesting that C79 was subsequently cleaved from CTFf. C96 was increased by TAPI-1 treatment to 1.81 ± 0.32 folds (*p* = 0.1436) though not significant, suggesting that ADAM10-generated C96 was further cleaved by ADAM17 (Fig. 3a, b, CNT + ADAM10 graphs). Consistently, TAPI-1 marginally increased CTFf to 1.22 ± 0.10 folds (*p* = 0.1251) in furin-transfected cells, while FI-2 significantly decreased CTFf with (0.49 ± 0.05 fold, *p* = 0.0017) and without TAPI-1 (0.55 ± 0.04 fold, *p* = 0.0036) (Fig. 3c, d), supporting that CTFf is generated by furin (Figs. 3c, d and 1e, f). In addition, TAPI-1 and FI-2 decreased C79 to 0.55 ± 0.09 fold (*p* = 0.0119) and 0.73 ± 0.11 fold (*p* = 0.1326) respectively, and the combination of TAPI-1 and FI-2 treatment further reduced C79 level to 0.44 ± 0.06 fold (*p* = 0.0034), suggesting the furin-ADAM17 sequential cleavage of CNTNAP2 (Fig. 3c, d). GI254023X is a potent ADAM10 inhibitor with a higher selectivity for ADAM10 over ADAM17. We expressed ADAM10 or ADAM17 in ADAM17-KO or ADAM10-KO cells, and then treated the cells with 0.1–10 μM GI254023X. 5 μM GI254023X inhibited ADAM10 cleavage (reduced C79 to 21.75 ± 7.58%, *p* < 0.0001; reduced C96 to 8.19 ± 3.44%, *p* < 0.0001, increased CNT-FL to 169.9 ± 9.20%, *p* = 0.0238), but not ADAM17 cleavage (brought C79 to 70.11 ± 10.25%, C96 to 106 ± 18.21%, CNT-FL to 112.5 ± 4.79%) of CNTNAP2 (Supplementary Fig. 4a, b). ADAM17-generated C96 was increased by 5 μM GI254023X treatment to 144 ± 22.68% (*p* = 0.1576), suggesting that C96 is further cleaved by ADAM10 (Supplementary Fig. 4c, d).

Taken together, these data indicated that CNTNAP2 undergoes complex sequential cleavages by furin and two α-secretases: ADAM10 and ADAM17. ADAM10 and ADAM17 share the same α-cleavage sites at L96 and I79 but show different site preferences, with ADAM17 predominantly at L96 and ADAM10 primarily at I79, resulting in C79 as the main CTF cleavage product of CNTNAP2 through sequential cleavages (Fig. 3g).

Next, we determined the relationship between α- and γ-secretase cleavages. In a separate study, we discovered that CNTNAP2 is cleaved by γ-secretase to generate the CNTNAP2 intracellular domain (CICD), and CNTNAP2-CICD expression improves ASD-related behaviors.^31^ L-685,458, a potent γ-secretase inhibitor (GSI), was applied to treat HEK cells co-transfected with CNTNAP2 and empty vector, ADAM10 or ADAM17. L-685,458 treatment significantly increased C79 levels in the empty vector (1.34 ± 0.06 folds, *p* = 0.0043), ADAM10 (1.97 ± 0.12 folds, *p* = 0.0013), and ADAM17 (1.78 ± 0.22 folds, *p* = 0.0227) overexpressing cells, indicating that the main α-cleavage product C79 is further cleaved by γ-secretase (Fig. 3e, f). Therefore, α-secretase cleavage is necessary for the subsequent γ-secretase cleavage to generate CNTNAP2-CICD (Fig. 3g).

### ASD-associated CNTNAP2 mutations impair the α-secretase processing of CNTNAP2

**Fig. 4:**
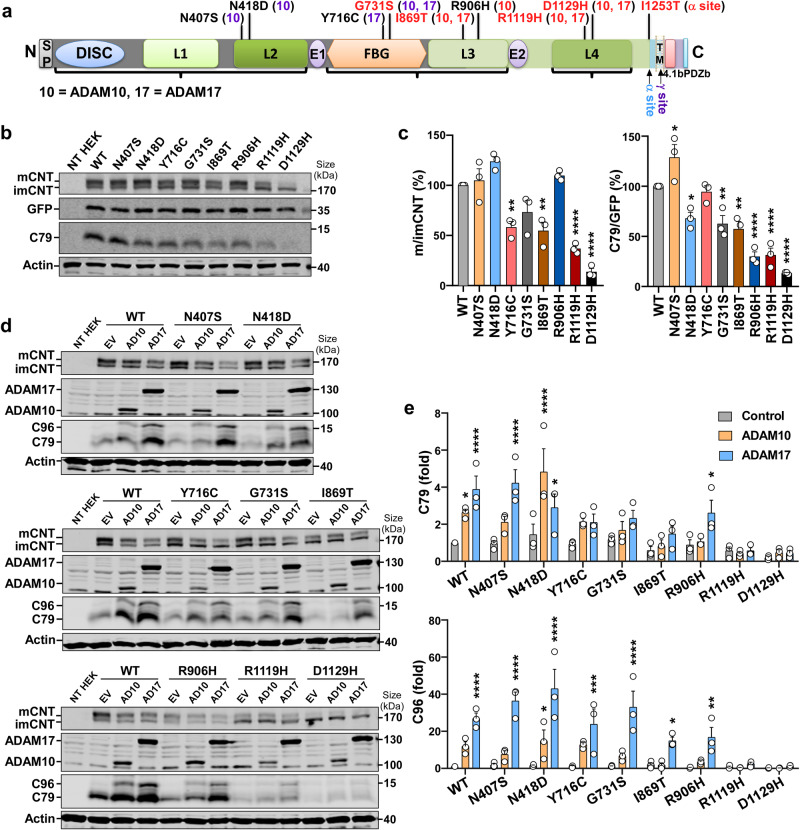
Pathogenic mutations affect CNTNAP2 processing. **a** Schematic of CNTNAP2 protein and locations of pathogenic mutations found in ASD patients. Mutations predicted deleterious or at conserved sites are noted in red. ADAM10/17-mediated α-cleavage affected by the mutations was summarized in the brackets (10 = ADAM10, 17 = ADAM17; slight affections are noted in purple, while severe impacts are marked in red). SP signal peptide, DISC discoidin-like domain, L1-4 four laminin A-like G domains, FBG fibrinogen-like domain, E1-2 two epidermal growth factor (EGF)-like domains, TM transmembrane domain; 4.1b, 4.1 binding domain; PDZb, PSD-95/Discs large/zona occludens-1 (PDZ) binding domain. Braces represent three lobes of CNTNAP2 protein. The figure is to scale. **b**, **c** CNTNAP2 mutations affected expression patterns of CNTNAP2 full-length and CTFs. CNTNAP2 plasmids were co-transfected with GFP into HEK cells. *n* = 3 independent experiments, ordinary one-way ANOVA followed by Dunnett’s multiple comparisons test, *p < 0.05; **p < 0.01; ****p < 0.0001. **d**, **e** Altered α-secretase cleavage in CNTNAP2 mutations. CNTNAP2 plasmids were co-transfected with empty vector (EV), ADAM10 (AD10), or ADAM17 (AD17) plasmids into HEK cells. Mutations showed variant cleavage patterns. Three blots show samples from the same experiment. The blots were processed in parallel and scanned together simultaneously. *n* = 3 independent experiments, two-way ANOVA followed by Dunnett’s multiple comparisons test (compare ADAM10 and ADAM17 with Control for each plasmid), row factor = mutation, column factor = ADAM10/17 overexpression. *p < 0.05; **p < 0.01; ***p < 0.001; ****p < 0.0001. All the results are expressed as mean ± SEM

Point mutations in *CNTNAP2* have been identified in ASD patients (Fig. 4a),^2^ however, the mechanism underlying the role of CNTNAP2 mutations in ASD pathogenesis is not clear. We discovered that I1253T at the α-secretase site I1253 (C-terminal I79 residue) inhibited the α-cleavage of CNTNAP2 to generate C79 (Fig. 2d, e, I79T corresponding to I1253T). Consistent with a previous report,^18^ we found that the mature/immature ratio of several mutant CNTNAP2 proteins differed from that of WT (WT, 100%; Y716C, 58.3 ± 5.6%, *p* = 0.0027; I869T, 54.86 ± 8.29%, *p* = 0.0012; R1119H, 36.95 ± 2.87%, *p* < 0.0001; D1129H, 13.78 ± 3.3%, *p* < 0.0001) (Fig. 4b, c). These mutations, particularly I869T (57.48 ± 7.35%, *p* = 0.0023), R906H (29.96 ± 4.66%, *p* < 0.0001), R1119H (31.47 ± 6.85%, *p* < 0.0001), and D1129H (13.34 ± 0.66%, *p* < 0.0001), reduced C79 levels (Fig. 4b, c). In the WT, C79 was increased to 2.64 ± 0.17 folds by ADAM10 or 3.88 ± 0.72 folds by ADAM17, and C96 was elevated to 11.87 ± 2.43 folds by ADAM10 or 26.92 ± 2.94 folds by ADAM17 (Fig. 4d, e). These pathogenic mutations generally affected α-secretase cleavage, especially the ADAM10 cleavage, with I869T, R1119H, and D1129H showing stronger inhibition on α-secretase cleavage. In R1119H, for example, C79 level was changed from 0.57 ± 0.20 in control to 0.43 ± 0.10 fold by ADAM10 and to 0.57 ± 0.17 fold by ADAM17, and C96 level was altered from 0.74 ± 0.49 to 0.39 ± 0.06 fold by ADAM10 and to 2.49 ± 0.86 folds by ADAM17, compared with the level of control in WT (Fig. 4d, e). Our results clearly demonstrate that the ASD-associated pathogenic mutations impair the α-secretase processing of CNTNAP2, leading to the reduction of CNTNAP2 CTF products.

### *Cntnap2*^-I1254T^ mice exhibit autism-like phenotypes and the C79 improves the phenotypes in vivo

**Fig. 5:**
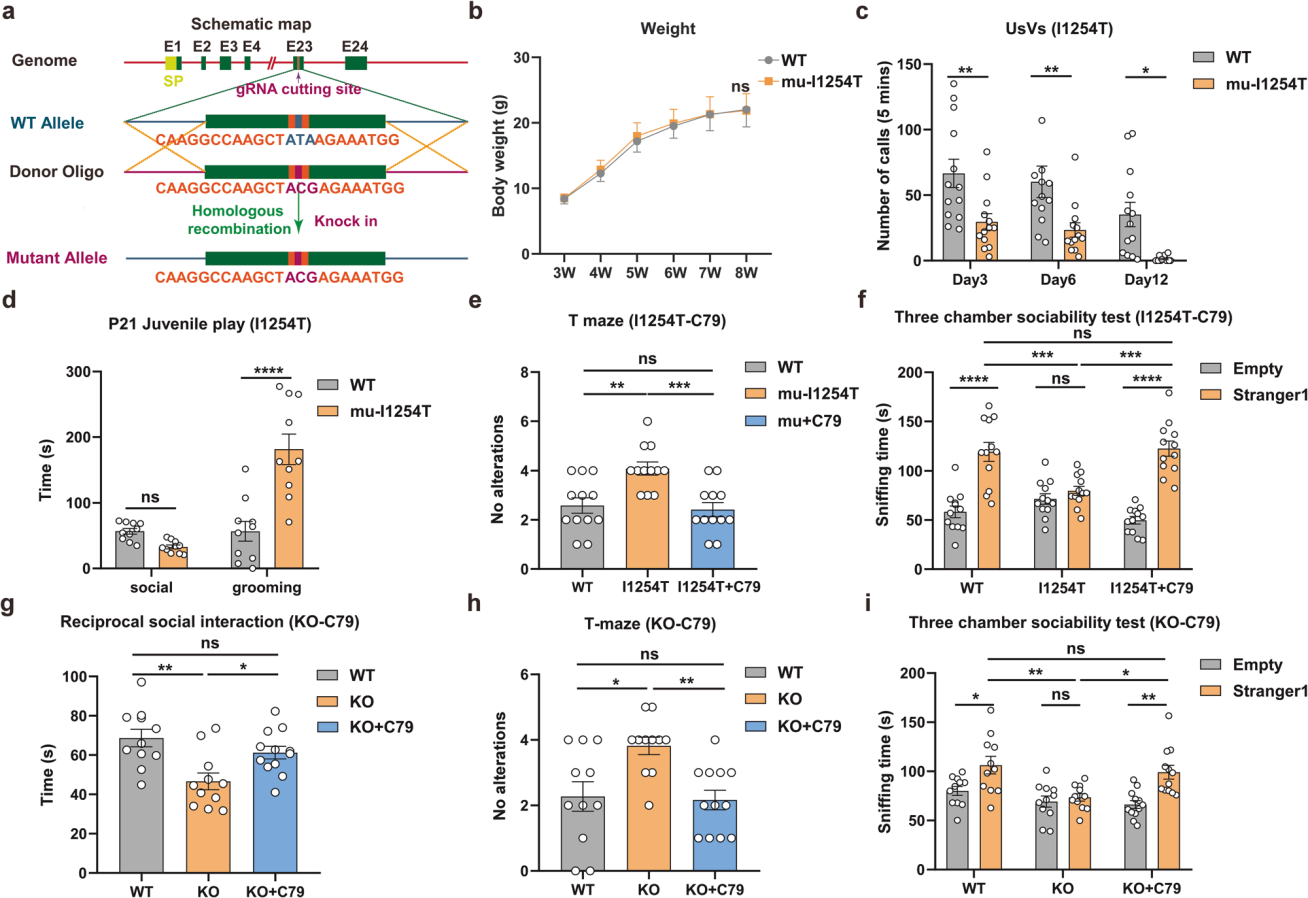
Impairment of α-secretase cleavage resulted in repetitive and social behavior abnormalities in mice. **a** Schematic representation of CRISPR/Cas9 knock-in method used to generate mutant I1254T mice by mutating “ATA” (Ile) to “ACG” (Thr) at the major α-cleavage site of CNTNAP2. **b** Body weight record of WT and I1254T mice. The weight of both mice was recorded daily, and no significant difference was found between mutants and WT mice (WT, *n* = 15; I1254T, *n* = 15). **c** Isolation-induced ultrasonic vocalizations (UsVs) test. The numbers of distress calls from infants of WT and mutants were detected at the age of P3, P6 and P12 (WT, *n* = 13; I1254T, *n* = 13). **d** Juvenile play test. Time involved in social interaction, as well as repetitive behaviors like grooming in WT and I1254T mutant mice were measured at the age of P21 when interacting with unfamiliar mice (*n* = 10 per group: male = 5, female = 5). **e**, **f** T maze and three chamber sociability tests performed at 7-week-old WT and mutants. I1254T mutation at the α-cleavage site increased repetitive behavior abnormalities in T maze (**e**) and decreased social interactions (**f**) in I1254T mutants, and C79 overexpression rescued the ASD-like behaviors (*n* = 12 per group: male = 6, female = 6). **g**–**i** Social and repetitive behavior tests in *Cntnap2*^*−/−*^ mice. C79 overexpression rescued the social interactions (**g**, **i**) and repetitive behaviors (**h**) in *Cntnap2*^*−/−*^ mice (*n* = 11: male = 6, female = 5 in WT and KO groups; *n* = 12: male = 6, female = 6 in KO + C79 group). Statistical significance was assessed by either one- or two-way ANOVA followed by Turkey or Bonferroni’s test. *p < 0.05; **p < 0.01; ***p < 0.001; ****p < 0.0001. All the results are expressed as mean ± SEM

To examine the effect of the essential α-secretase cleavage of CNTNAP2 on autism-like phenotypic behaviors, we generated mutant I1254T knock-in mice (*Cntnap2*^-*I1254T*^) using the CRISPR/Cas9 technique to express the I1254T mutant CNTNAP2 protein, corresponding to the pathogenic I1253T mutation in ASD patients (Fig. 5a). All mice were bred through crossing heterozygous *Cntnap2*^-*I1254T*^ and further confirmed by genotype sequencing. The pups were born at a Mendelian ratio, and there was no gender difference of pups (Supplementary Table 2 and Supplementary Fig. 5). *Cntnap2*^-*I1254T*^ mice grew as well as control mice during the 8 weeks of study period. Body weight was monitored from 3 to 8 weeks of age, and there was no difference in body weight between the mutant *Cntnap2*^-*I1254T*^ mice and control WT mice (Fig. 5b).

The mice were tested with the isolation-induced ultrasonic vocalizations (UsVs) at the ages of 3, 6 and 12 days, in which the distress calls emitted by separated pups and the infant-mother vocal communications were considered as ASD-like behavior.^3,32^ *Cntnap2*^-*I1254T*^ mice emitted a significantly lower number of distress calls than WT mice at different ages (Fig. 5c). In the Juvenile Play test where the social interaction and repetitive behaviors between different pairs of unfamiliar mice was recorded, the *Cntnap2*^-*I1254T*^ mice showed increased repetitive behaviors like grooming (Fig. 5d).

To confirm the autism-like behaviors was due to the impaired α-cleavage of CNTNAP2 at ADAM10 site, the adeno-associated virus (AAV) vector expressing CNTNAP2-C79 protein (AAV-C79) and AAV-EGFP (as the control) were generated and injected into the medial prefrontal cortex (mPFC) of the mutant (*Cntnap2*^-*I1254T*^) and CNTNAP2-KO mice (*Cntnap2*^*−/−*^) at the age of 3 weeks (Supplementary Fig. 6a). AAV-C79 injection significantly increased the C79 protein level in mPFC (Supplementary Fig. 6b). In an open-field test, *Cntnap2*^-*I1254T*^ mice displayed locomotor activity similar to that of the WT mice (Supplementary Fig. 7a), while the *Cntnap2*^*−/−*^ mice exhibited hyper locomotion compared with the WT mice (Supplementary Fig. 7b). In addition, *Cntnap2*^-*I1254T*^ mice injected with AAV-EGFP displayed an increased number of no alterations, representing an ASD-like repetitive behavior (Fig. 5e). In the three-chamber social interaction test, all mice showed no preference for each chamber in the habituation phase (Supplementary Fig. 8a, b), and *Cntnap2*^-*I1254T*^ mice did not show a significant social preference for strangers when compared to the highly social-interactive WT mice (Fig. 5f). However, the expression of C79 significantly reduced the number of no alterations of *Cntnap2*^-*I1254T*^ mice in T maze test (Fig. 5e) and increased their social interactions with unfamiliar mice (Fig. 5f) at the age of 7 weeks, indicating that C79 expression improved autism-like phenotypes in the mutant mice. Similarly, C79 expression also rescued the deficiency in repetitive and social behaviors of *Cntnap2*^*−/−*^ mice (Fig. 5g–i). Taken together, these data demonstrate that the mutation of α-cleavage site in CNTNAP2 leads to autism-like repetitive and social behavior abnormalities, and restoring α-cleavage product C79 expression improves autism-like phenotypes.

## Discussion

The α- (ADAM10 and ADAM17), β- (BACE1) and γ- (Presenilin, Nicastrin, Aph1, Pen2) secretases play essential roles in processing APP,^27,33^ and dysregulation of these key enzymes have been shown to facilitate Alzheimer’s pathogenesis.^34–38^ Our study revealed that CNTNAP2 protein undergoes a complex proteolytic processing pathway in which CNTNAP2 is sequentially cleaved by furin, α-, and γ-secretases. Furin cleaves mature CNTNAP2 within the L3 domain to yield CTFf, and CTFf is subsequently cleaved by α-secretase ADAM10/17. ADAM10 and ADAM17 share the same cutting sites at I79 and L96, but have different site preference: ADAM10 predominantly cleaves CNTNAP2 at I79 to generate CTFα1 C79, whereas ADAM17 preferably cleaves CNTNAP2 at L96 to produce CTFα2 C96. C96 is further processed by α-secretase to generate C79. The presenilins-dependent γ-secretase subsequently cleaves C79 to generate CNTNAP2-CICD. ASD-associated pathogenic CNTNAP2 mutations impair the α-cleavage to generate C79, and the mutation of α-cleavage site in CNTNAP2 leads to ASD-like repetitive and social behavior abnormalities. Finally, restoring α-cleavage product C79 expression improves autism-like phenotypes.

The current study demonstrates that CNTNAP2-C79 generated by α-secretase cleavage is absolutely required for the subsequent γ-secretase cleavage to yield the CNTNAP2-CICD. We recently reported that CICD enters the nucleus and functions as a transcription factor to improve autism-related behaviors.^31^ Therefore, disruptions in the α-cleavage of CNTNAP2, such as by pathogenic mutations shown in Fig. 4, may affect the downstream CNTNAP2 signaling pathway.

ADAM10 is widely expressed in the brain and functions as a major protease in the central nervous system.^39^ It is synthesized in the rough endoplasmic reticulum (ER) and undergoes maturation as it is transported through the Golgi apparatus. During maturation, the prodomain of ADAM10, which keeps the enzyme in an inactive state, is removed. This removal of the prodomain is a crucial step in activating ADAM10 and allowing it to exert its enzymatic functions. As a prominent α-secretase enzyme, it is primarily recognized for its involvement in APP processing to regulate the production of Aβ. In the context of AD, the activation of ADAM10 has also been shown to elevate the levels of soluble triggering receptor expressed on myeloid cells-2 (sTREM2) by influencing the extracellular domain of TREM2. The sTREM2 is known to possess neuroprotective properties as it enhances the degradation of Aβ plaques.^40^ ADAM10 also plays a significant role in the shedding of proteins that are crucial for brain development, including cadherins, ephrins, and Notch receptors. Notch receptors are key regulators of cell proliferation, cell fate decisions and differentiation. ADAM10 is involved in the proteolytic cleavage of Notch receptors, which is a critical step in the activation of Notch signaling and influences cell fate decisions during neurogenesis and brain development. *Adam10*-KO mice die at embryonic day 9.5 due to disruptions of the Notch signaling.^41^ Both conditional *Adam10*-KO mice^42,43^ and *Cntnap2*-KO mice^3,44^ are viable but have seizures, learning deficits, neuronal migration abnormalities, and aberrant spine morphology, implicating the significance of CNTNAP2 as a critical substrate cleaved by ADAM10. Notably, we found that the CNTNAP2-CICD or C79 is sufficient to rescue the behavioral deficits in the *Cntnap2*^*−/−*^ knockout mouse, suggesting the cleaved C79 is the key molecule for CNTNAP2’s physiological functions. Consistent with the pathogenic effect of I1253T mutation in ASD patients, *Cntnap2*^*-I1254T*^ knock-in mice displayed characteristic autism-like phenotypes that can be rescued by restoring C79 expression, highlighting the essential function of C79 in CNTNAP2 across species.

Post-translational modifications play a crucial role in determining and regulating a protein’s structure, localization and functions. APP undergoes N- and O-glycosylation, phosphorylation, sulfation, palmitoylation, ubiquitination and sumoylation following its synthesis. It is believed that these post-translational modifications are required for the cleavage of APP within an intracellular secretory pathway.^45^ APP modifications also play a crucial role in its trafficking, which is mutually regulated and collectively contributes to the regulation of APP processing and Aβ generation.^45^ Similar to APP processing, our study has revealed that the cleavage of CNTNAP2 by the proteases occurs only in its mature form. CNTNAP2 undergoes N-linked glycosylation during its maturation.^10,11,18^ Disruptions in glycosylation could affect CNTNAP2 trafficking, localization, interaction, and degradation, particularly given that mature and immature forms of CNTNAP2 are degraded in different degradation pathways.^21^ We and others found that ASD pathogenic mutations I869T, R1119H, and D1129H, have reduced mature/immature ratios and trafficking deficits, leading to retention in the endoplasmic reticulum.^18^ As expected, our study found that these mutations affected α-secretase cleavage and reduced C79 generation. Future studies are warranted to further examine CNTNAP2’s post-translational modifications, which could be critical for CNTNAP2’s proteolytic processing. Although G731S and R906H showed relatively normal maturation rates, ADAM10-dependent α-secretase cleavage was largely reduced, resulting in less C79 generation. The impaired cleavage is most prominent in I1253T, which resides precisely at the α-cleavage site. Although pathogenic mutations may contribute to ASD pathogenesis by different mechanisms, one common consequence is the reduced C79 level.

CNTNAP2 is processed by MMP9, and the released ectodomain regulates Ca^2+^ homeostasis and reduces neuronal network synchrony.^30^ MMPs and ADAMs, both belonging to metzincin metalloproteinases, are widely expressed in the nervous system^46^ and sometimes share substrates. For instance, syndecan-1 and syndecan-4 are cleaved by both MMP9 and ADAM17 close to the cell membrane.^47,48^ Our study clearly identified two α-cleavage sites by ADAM10 and ADAM17, and our results from the KO cell lines unequivocally demonstrated that CNTNAP2 is mainly cleaved by ADAM10. Additionally, this study is the first to show the complete CTF pattern of CNTNAP2. Our finding that CNTNAP2 undergoes complex proteolytic processing provides novel clues for in-depth studies of the CNTNAP2 signaling pathway. Future studies are warranted to reveal the functions of cleaved ectodomains and CTFs. Here we show that C79 generated by α-secretase is a key molecule of CNTNAP2 signaling, the disruption of which may contribute to ASD pathogenesis. Furthermore, we found that restoring C79 expression improves autism-like phenotypes in the *CNTNAP2*^-I1254T^ mutant mice and *CNTNAP2*^*−/−*^ knockout mice. Therefore, replenishment of CNTNAP2-C79 or CNTNAP2-CICD to the physiological level could be a potential avenue to treat ASD.

## Materials and methods

### Plasmid constructs

pRK5-CNTNAP2 encodes full-length human CNTNAP2 protein 1-1331 aa, with an HA tag after the signal peptide and a Flag tag fused to the C-terminal. pRK5M-ADAM10 (Addgene plasmid # 31717; http://n2t.net/addgene:31717; RRID:Addgene_31717) and pRK5M-ADAM17 (pRK5M-TACE, Addgene plasmid # 31714; http://n2t.net/addgene:31714; RRID:Addgene_31714) were gifts from Rik Derynck.^49^ Human furin cDNA was cloned into pcDNA4/myc-HisA (pz) expression vector (Invitrogen Cat# V86320). 178myc (M + the last 177 aa), 108myc, 91myc (M + the last 90 aa) with C-terminal 6myc tag were cloned into the pz vector. CNTNAP2 protein ladders with C-terminal Flag tag, C96 (M + the last 95 aa) and C79 (M + the last 78 aa), were cloned into pz vector using BamH1 + Xho1. WT plasmid (Fig. 2) containing the last 108 aa of CNTNAP2 with C-terminal Flag tag was cloned into pz vector using BamH1 + Xho1. Mutant plasmids were generated based on the WT plasmid by PCR-based site-directed mutagenesis using primers in Supplementary Table 3. CNTNAP2 WT and mutant plasmids in Fig. 4 were kindly provided by Dr. Laurence Goutebroze.^18^

### Generation of knockout cell lines by CRISPR/Cas9

HEK-ADAM10-KO and HEK-ADAM17-KO cell lines were generated by Gemple biotechnology (China). The main protocols are as follows. First, single guide RNAs (sgRNAs) were synthesized using GeneArt Precision gRNA Synthesis Kit (Invitrogen Cat# A29377), two sgRNAs targeting ADAM10 exon 2 and two sgRNAs targeting ADAM17 exon 3. Next, sgRNA/Cas9 ribonucleoprotein was delivered into HEK cells via electroporation (Neon Transfection System, Invitrogen). 3 days later, transfected cells were harvested for gDNA extraction, PCR, and Sanger Sequencing. Single KO cell clones were isolated and expanded, and then sequenced again. Finally, HEK-ADAM10-KO (+1 bp/-17bp) and HEK-ADAM17-KO (-14bp/-14bp) cell lines were verified by Sanger Sequencing and Western blot.

### Cell culture, transfection, and treatment

HEK, HEK-ADAM10-KO, HEK-ADAM17-KO, and N2a cells were cultured in high-glucose Dulbecco’s modified Eagle medium (Cytiva Cat# SH30243.01) containing 10% fetal bovine serum (Gibco Cat# 12483020) and 100 U/mL Penicillin-Streptomycin (Gibco Cat# 15140122). Cells were maintained at 37 °C in a 5% CO2 incubator. Transfection was performed with Polyethylenimine for HEK cells and Lipofectamine 2000 Reagent (Invitrogen Cat# 11668019) for N2a cells following the manufacturer’s instructions. For the inhibitor treatment, HEK cells were transfected in a 6 cm master plate and then split into 4 × 35 mm plates 4 h after transfection, maintained overnight (16 h) before adding inhibitors. Cells were treated with different inhibitors for 24 h before harvested. ADAM17 inhibitor TAPI-1 (Cat# CAS 171235-71-5), Furin inhibitor 2 (Cat# SCP0148), ADAM10 inhibitor GI254023X (Cat# SML0789) and γ-secretase inhibitor L-685,458 (Cat# L1790) were purchased from Sigma-Aldrich.

### Immunoblot analysis

Cells were lysed in RIPA lysis buffer supplemented with the cOmplete Protease Inhibitor Cocktail (Roche). Cell lysates were resolved on 7.5% Tris-glycine or 16% Tris-tricine (for CTF) SDS-PAGE and transferred to nitrocellulose membranes. Membranes were blocked in 5% non-fat milk in phosphate-buffered saline (PBS) for 1 h at room temperature and then blotted with primary antibodies overnight at 4 °C with shaking. On the next day, membranes were blotted with IRDyeTM 680-labeled (1: 10000, LI-COR Biosciences Cat# 926-68070, RRID: AB_10956588) or IRDyeTM 800CW-labeled (1: 10000, LI-COR Biosciences Cat# 926-32211, RRID: AB_621843) secondary antibodies for 1 h at room temperature and then visualized using the LI-COR system. The following rabbit antibodies were used: anti-FLAG (1: 2000, Abcam Cat# ab1162, RRID: AB_298215) was used to detect CTF with Flag tag; N63 (1:1000, targeting 63-95 aa of CNTNAP2 at the N-terminal) and C22 (1:1000, targeting the last 22 aa of CNTNAP2 at the C-terminal), generated by our lab,^21^ were used to detect CNTNAP2 full-length and CTF; anti-ADAM10 (1:1000, Abcam Cat# ab124695, RRID: AB_10972023) and anti-ADAM17 (1:1000, Santa Cruz Biotechnology Cat# sc-390859) were used to detect endogenous ADAM10 and ADAM17; anti-GFP (1:1000, Santa Cruz Biotechnology Cat# sc-8334, RRID: AB_641123) was used to detect GFP. The following mouse antibodies were used: anti-HA (1:50, 12CA3, generated in our lab) was used to detect CNTNAP2 full-length with HA tag; anti-myc (1:20, 9E10) was used to detect transfected ADAM10, ADAM17, and furin with C-terminal myc tag; anti-Actin (1:50000, Sigma-Aldrich Cat# A1978, RRID: AB_ 476692) was applied for Actin detection.

### Immunocytochemistry

HEK and N2a cells were transfected and then plated on coverslips pre-coated with Poly-D-Lysine (Sigma-Aldrich Cat# P7886). 24 h later, cells were fixed with 4% paraformaldehyde in PBS for 10 min at room temperature and then washed 3 times with PBS. After blocking with 5% Bovine Serum Albumin (BSA) in PBS for 1 h at room temperature, cells were incubated with primary antibodies overnight at 4 °C in a humidity chamber. On the next day, cells were washed 3 times with PBS and then incubated with secondary antibodies for 1 h at room temperature in a humidity chamber protected from the light. After 3 washes with PBS, coverslips were mounted using Fluoromount-G with DAPI (Invitrogen Cat# 00-4959-52) and air-dried in the dark. CNTNAP2 was detected by anti-CNTNAP2 targeting extracellular 1001-1142 aa (1: 200, Sigma-Aldrich Cat# HPA002739, RRID: AB_1078545) and 488-Donkey anti-Rabbit IgG (1: 500, Thermo Fisher Scientific Cat# A-21206, RRID: AB_2535792). Myc-tagged ADAM10, ADAM17, and furin were detected by anti-myc (1:200, 9E10, generated in our lab) and 568-Goat anti-Mouse IgG (1: 500, Thermo Fisher Scientific Cat# A-11031, RRID: AB_144696). Antibodies were prepared with 1% BSA in PBS. The staining results were viewed under the 63x oil lens of the Zeiss fluorescence microscope. Images taken under the Apotome mode were used to reflect the membrane expression of CNTNAP2 in Fig. 1g. Corresponding conventional fluorescent images (Supplementary Fig. 2) were quantified using Zeiss software to investigate the cleavage. 10 cells for each transfection group were randomly chosen from two independent experiments (5 cells from each). The exposure time for each channel in HEK and N2a cells was as follows: DAPI 8 ms, EGFP (green) 15 ms, and AF568 (red) 60 ms. The area of individual cell was outlined by the Spline Contour graphic. Then, the mean intensity value for the outlined cell area was automatically produced in the Measure tab in Zeiss software. The average intensity of 10 cells from the vector group was used to normalize the value across all transfection groups in percentage.

### In vitro cleavage and mass spectroscopy

Two synthetic peptides (10 μM, Bio Basic Inc.), peptide 1 (the last 108-84 aa, MSSATDPWHLDHLDSASADFPYNPG) and peptide 2 (the last 96-72 aa, LDSASADFPYNPGQGQAIRNGVNR N), were incubated with or without recombinant human ADAM17 (0.5 μM, R&D system Cat# 930-ADB) in the assay buffer (25 mM Tris, 2.5 μM ZnCl2, pH 9.0) for 24 h at 37 °C. The resulting solution was sent to mass spectroscopy (Proteomics Core Facility, UBC, Canada) to identify the cleavage sites.

### Immunoprecipitation and N-terminal sequencing

30 × 10 cm plates of HEK cells were co-transfected with pRK5-CNTNAP2 and pRK5M-ADAM17 and then treated with 5 μM MG132 (Sigma-Aldrich Cat# C2211) and 20 nM γ-secretase inhibitor L-685,458 for 16 h before harvested to increase CTF. Cells were lysed using RIPA buffer and precleared with Sepharose 4B (Sigma-Aldrich Cat# 4B200) for 1 h at 4 °C. Precleared lysates were incubated with anti-FLAG M2 affinity gel (Sigma-Aldrich Cat# A2220, RRID: AB_10063035) overnight at 4 °C with rotating. On the next day, unbound proteins were washed out, and precipitated proteins were eluted via boiling in 1 x sample buffer. Eluted proteins were resolved on 16% Tris- tricine SDS-PAGE and transferred to PVDF membranes. Membranes were stained with Coomassie blue to visualize the precipitated CTF and then sent for N-terminal sequencing (The Protein Facility of the Iowa State University).

### Mice

*Cntnap2* mutant and wild-type (WT) mice were generated through crossing mutant heterozygous *Cntnap2* mutant mice, and offspring were born with the expected Mendelian frequencies. The genotypes of the mice were confirmed by sequencing the DNA extracted from tail tissues with forward primer CNTNAP2 5′-TGGATACTACAAGACAATAGCAAG and reverse primer CNTNAP2 5′-ACCTTGATGAGGCTATAATTGAAC. 3–4 mice of the same sex were raised in each cage with 12-h light and dark cycle, and water and food were available at all day. For each behavioral test, 10-15 mice injected with either AAV-EGFP or AAV-C79 were included. All behavioral tests were performed and analyzed with the ANY-maze automated system. Ultrasonic Vocalizations were analyzed with Sonotrack sound analysis and synthesis software for laboratory animals. All procedures regarding the care and use of animals were approved by the Ethics Committee of Wenzhou Medical University. All animal procedures were performed in accordance with the Wenzhou Medical University Animal Research Center protocols.

### Ultrasonic Vocalization (UsV) Test

To detect distress calls emitted by separated infants, pups at different ages of 3, 6, and 12 days were transferred into 400 × 300 × 220 mm cardboard boxes that were outfitted with UsV recording equipment for 5 min. The room temperature was kept at 21 °C to prevent temperature-related variable factors. The total number of distress calls within 5 min was recorded by Sonotrack software.

### Juvenile play test

The animals were allowed to habituate for a minimum of 1 h prior to the start of behavior test. Mice aged P21 were placed in a new cage and kept for 10 min with a genotype- and sex-matched stranger mouse. The mice performing social interactions such as nose-to-nose sniffing and nose-to-anal sniffing, as well as repetitive behaviors such as grooming were measured by human observer.^3,32^

### T maze spontaneous alternation

In brief, 7-week-old mice were given proper acclimatization in the test room for a minimum of 1 h. Then, mice were situated at the base of a T-maze equipment to explore either the right or left arm of the maze for 10 consecutive trials. The number of no spontaneous alternations of mice were counted and recorded by the observer.

### Reciprocal social interaction

As previously described,^50^ 7-week old mice were tested in this experiment. After 1 h of acclimatization, the mice were habituated in a social box for 10 min. Then, an unfamiliar mouse of the same genotype, age and sex was introduced into the social box, and their interactions were recorded by video for 10 min. The interactions, including nose-to-nose, nose-to-genital communication, and body contacts, were measured.

### Open field test

Briefly, the whole experiment took place in a square arena (50 × 50 cm), which was mounted within specially designed sound attenuating shells constructed of polypropylene, regular and expanded PVC. All mice were placed in the center of the open field arena and allowed to freely move for 15 min while being tracked by an automated tracking system.

### Three chamber social interaction test

The social interaction test was performed as previously described.^3,7^ Briefly, after 1-h habituation, mice were placed in a clear Plexiglas box to explore all three divided chambers for 10 min to test locomotor activity. Then the mice were allowed to freely interact with either an empty wire cup (positioned in one side chamber) or with a wire cup with a stranger mouse inside (positioned in the opposite side chamber). The time of social interaction was measured and analyzed with an ANY-maze automated system.

### Statistics

All the results are expressed as mean ± standard error of the mean (SEM). Replicate numbers of independent experiments, cells, or animals are noted in the figure legends. GraphPad Prism was used for statistical analyses and for creating quantification figures. Comparison between two groups was analyzed by the 2-tailed Student’s *t*-test. Multiple comparisons were analyzed by one-way or two-way ANOVA followed by post hoc multiple comparisons test. *P* < 0.05 was considered statistically significant.

### Supplementary information

Supplementary materials

## Data Availability

The data that support the findings of this study are available from the corresponding author upon request.
