# Supplementary material for: Contactin-associated protein-like 2 (CNTNAP2) mutations impair the essential α-secretase cleavages, leading to autism-like phenotypes
Source: Signal Transduct Target Ther. 2024 Mar 1;9:51. doi: 10.1038/s41392-024-01768-6 (PMC10904759; doi:10.1038/s41392-024-01768-6)
Supplement: Supplementary file 1 — Supplementary materials [file 41392_2024_1768_MOESM1_ESM.docx]

**Supplementary Materials for**

***Contactin-associated protein-like 2 (CNTNAP2)* mutations impair the essential α-secretase cleavages, leading to autism-like phenotypes**

**Qing Zhang^1,2^†, Mengen Xing^1^†, Zhengkai Bao^1^†, Lu Xu^1^, Yang Bai^1^, Wanqi Chen^1^, Wenhao Pan^1^, Fang Cai^1^, Qunxian Wang^4^, Shipeng Guo^4^, Jing Zhang^5^, Zhe Wang^3^, Yili Wu^1^, Yun Zhang^3*^, Jia-Da Li^5*^, Weihong Song^1,2,3,4*^**

Correspondence to: Weihong Song, [weihong@wmu.edu.cn](mailto:weihong@wmu.edu.cn); Yun Zhang, [zhangyun@xwhosp.org](mailto:zhangyun@xwhosp.org) and Jia-da Li, [lijiada@sklmg.edu.cn](mailto:lijiada@sklmg.edu.cn)

**This PDF file includes:**

Supplementary Figure 1 to 8

Supplementary Tables 1 to 3

**Supplementary Figures**


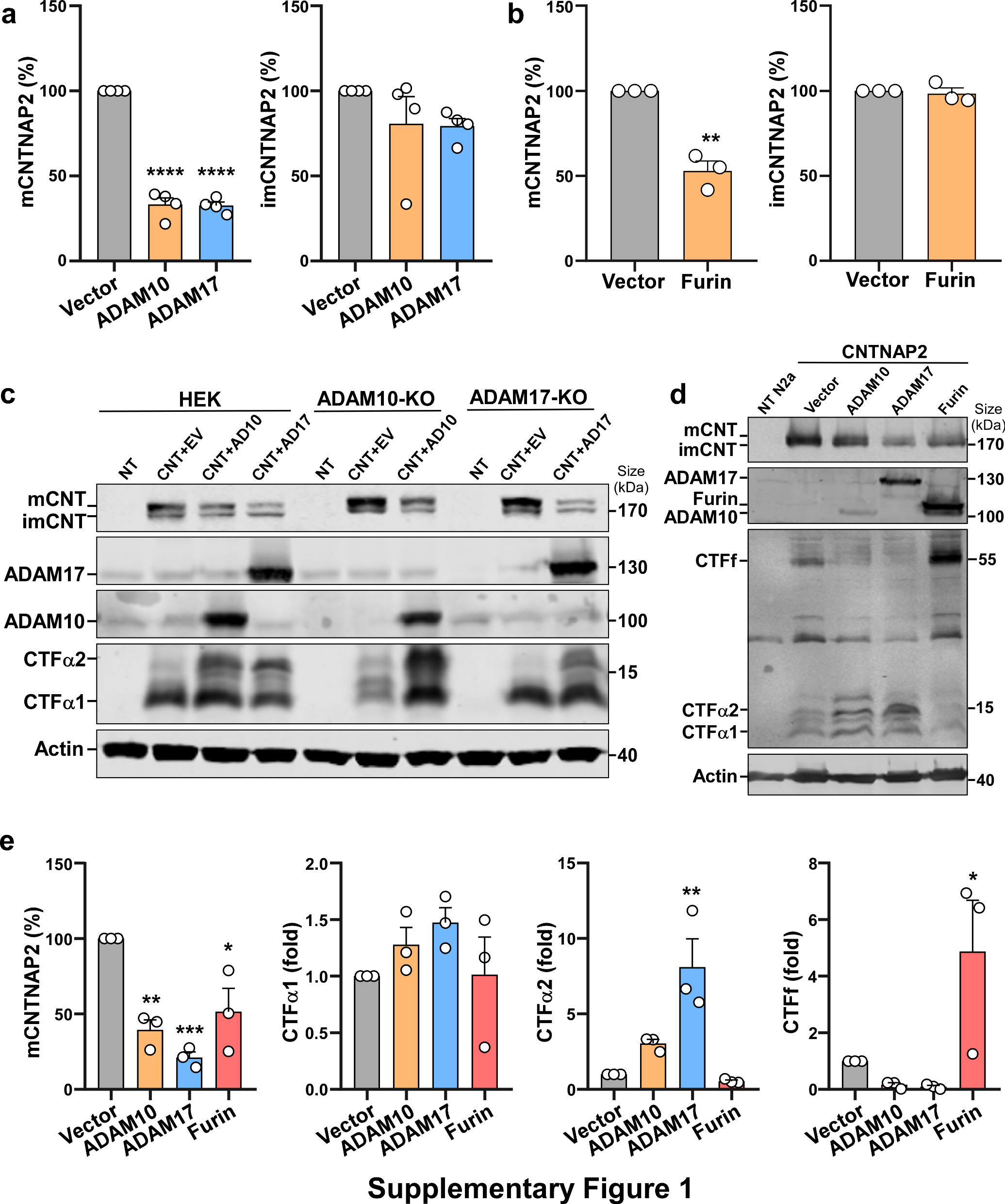


**Supplementary Figure 1. CNTNAP2 is cleaved by α-secretase and furin (related to Figure 1). a** Glycosylated mature CNTNAP2 was cleaved by ADAM10 and ADAM17. Immature intracellular CNTNAP2 was not affected by ADAM10 and ADAM17. n = 3 independent experiments, ordinary one-way ANOVA followed by Dunnett’s multiple comparisons test, ****p < 0.0001. **b** Mature CNTNAP2 was reduced by furin, while immature CNTNAP2 was not affected. n = 3 independent experiments, unpaired t-test, **p < 0.01. **c** ADAM10 is the major α-secretase that generates CTFα1. CNTNAP2 was co-transfected with vector, ADAM10, or ADAM17 into HEK, ADAM10-knockout (KO), or ADAM17-KO cells. **d, e** CNTNAP2 processing in N2a cells. CNTNAP2 was co-transfected with vector, ADAM10, ADAM17, or furin into N2a cells. All the co-transfected plasmids were detected by the C-terminal myc tag. n = 3 independent experiments, ordinary one-way ANOVA followed by Dunnett’s multiple comparisons test, *p < 0.05; **p < 0.01; ***p < 0.001; ****p < 0.0001. All the results are expressed as mean ± SEM.


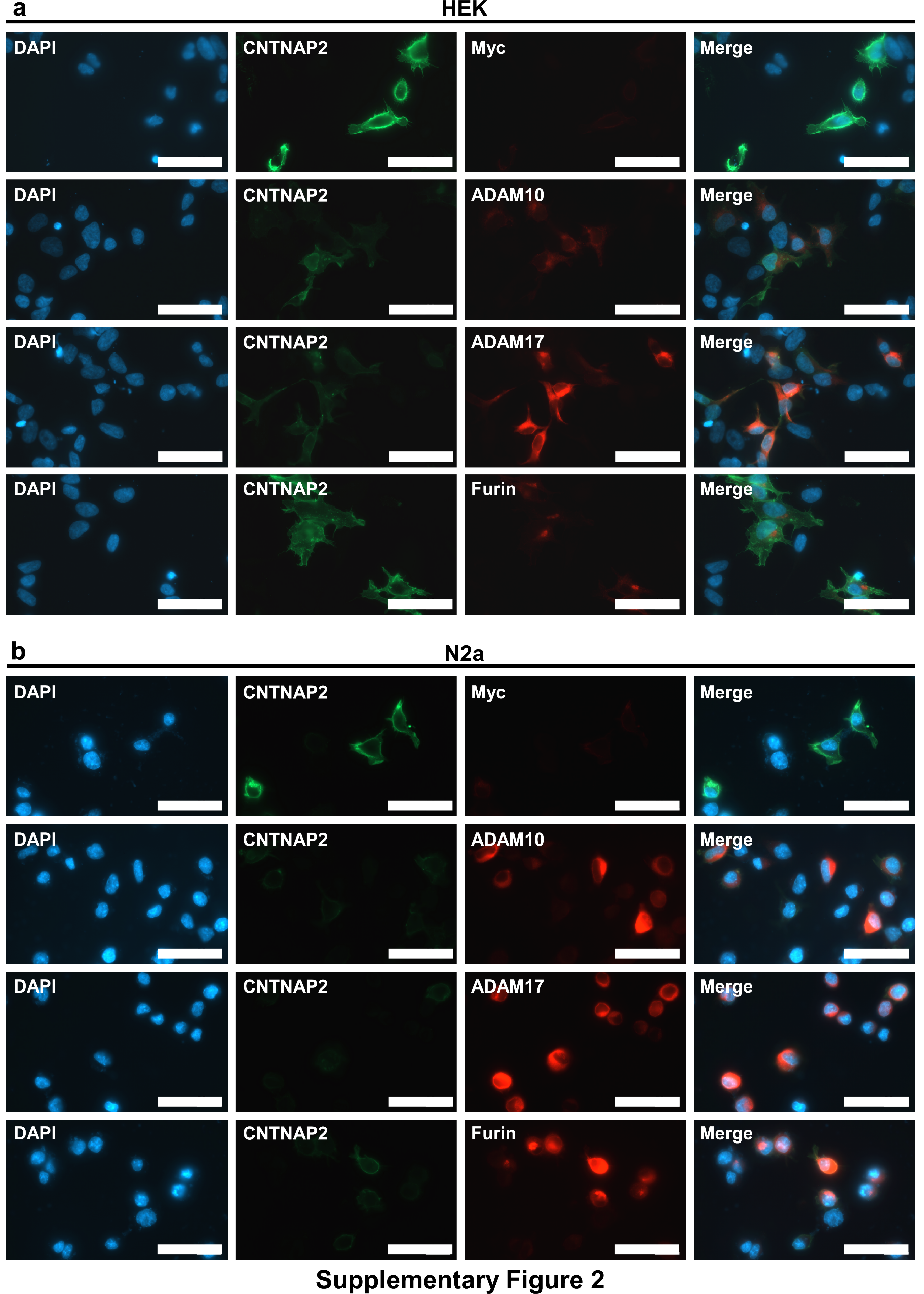


**Supplementary Figure 2. Conventional fluorescence images (related to Figure 1).** Fluorescence images were used for the quantification of immunocytochemistry in HEK (a) and N2a (b). CNTNAP2 mean intensity of transfected cells was measured using the measurement function in ZEISS. Scale bar = 50 μm.


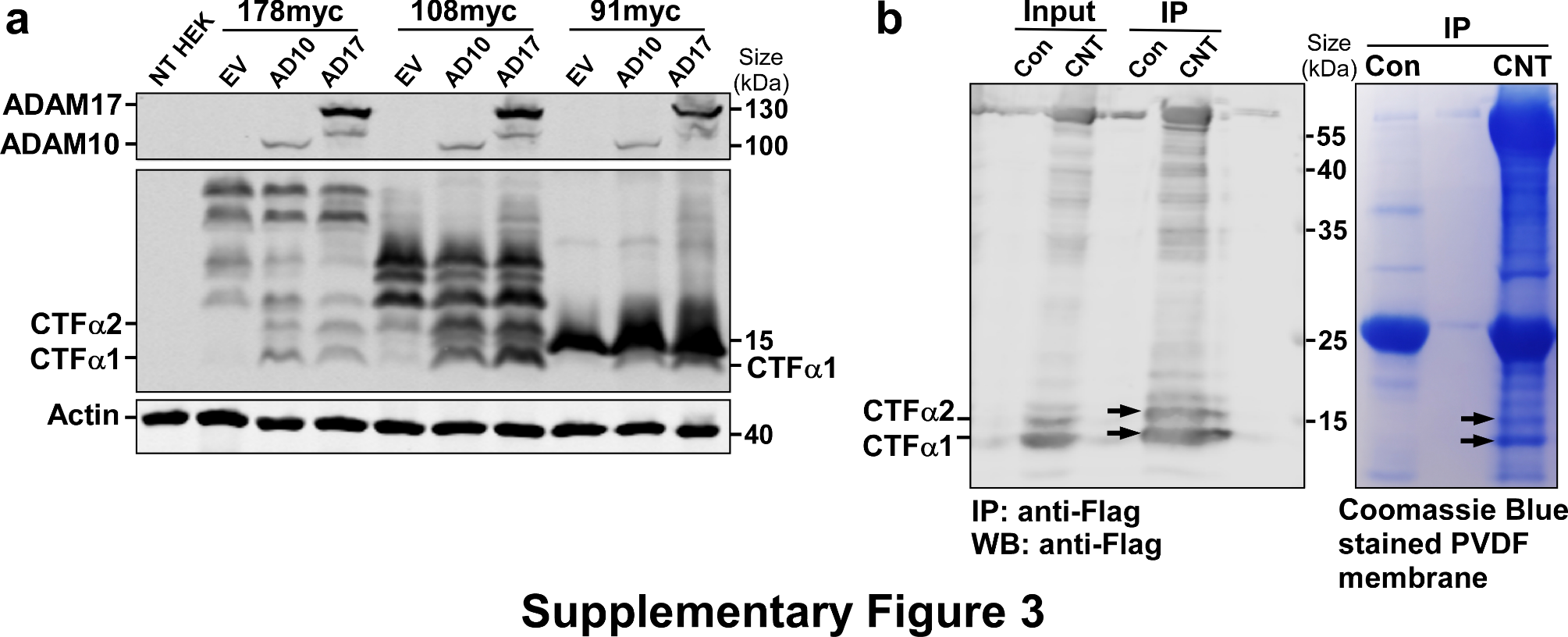


**Supplementary Figure 3. Identification of α-secretase cleavage site (related to Figure 2). a** Narrow down the cleavage sites. Three shorter fragments of CNTNAP2 were generated: pz-CNTNAP2-178myc, pz-CNTNAP2-108myc, pz-CNTNAP2-91myc, containing the last 177 aa, 108 aa, 90 aa of CNTNAP2, respectively. CNTNAP2 fragments were co-transfected with empty vector (EV), ADAM10 (AD10), or ADAM17 (AD17). **b** N-terminal sequencing. Immunoprecipitation (IP) was performed using anti-FLAG M2 affinity gel to get precipitated CTFα1 and CTFα2 from HEK cells co-transfected with pRK5-CNTNAP2 and pRK5M-ADAM17. Western Blot (WB) was performed with the anti-FLAG antibody to show the successful IP. Black arrows pointed out CTFα1 and CTFα2 on the Coomassie Blue stained PVDF membrane, which was sent to N-terminal sequencing.


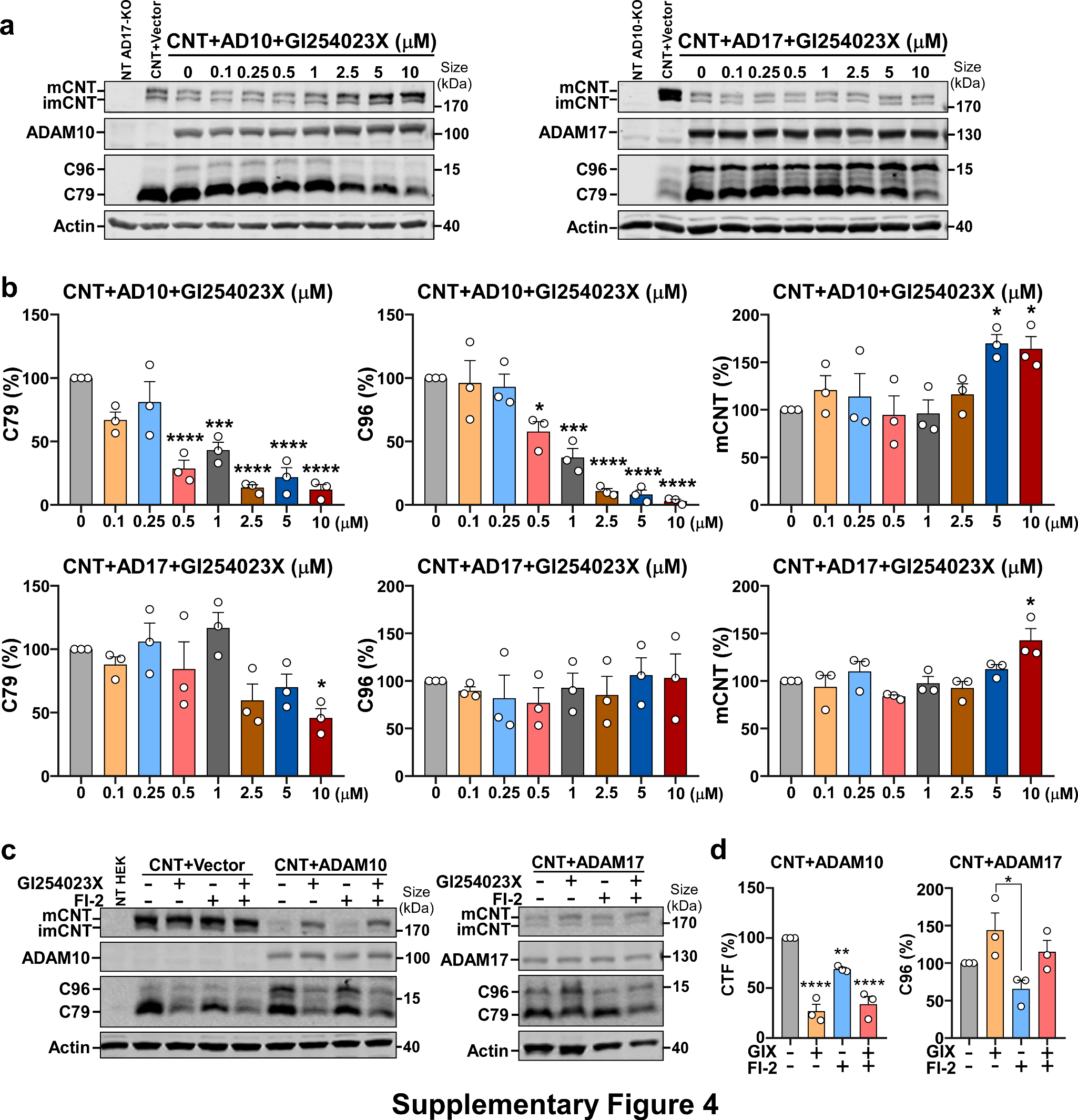


**Supplementary Figure 4. Sequential cleavage of furin and α-secretase (related to Figure 3). a, b** 5 uM GI254023X selectively inhibited ADAM10 cleavage of CNTNAP2. To screen out the selective dose for ADAM10 inhibition, CNTNAP2 was co-transfected with ADAM10 or ADAM17 into ADAM17-KO or ADAM10-KO cells, followed by treatment with GI254023X of increasing dosages. n = 3 independent experiments, ordinary one-way ANOVA followed by Dunnett’s multiple comparisons test, *p < 0.05; ***p < 0.001, ****p < 0.0001. **c, d** ADAM17 generated C96 was further cleaved by ADAM10. Same as in Fig. 3A, Control, ADAM10, and ADAM17 transfected cells were treated with 5 uM GI254023X and 10 uM FI-2. n = 3 independent experiments, ordinary one-way ANOVA followed by Tukey’s multiple comparisons test, *p < 0.05; **p < 0.01, ****p < 0.0001. P values stand for comparisons with the Control group unless noted by the brackets. All the results are expressed as mean ± SEM.

**
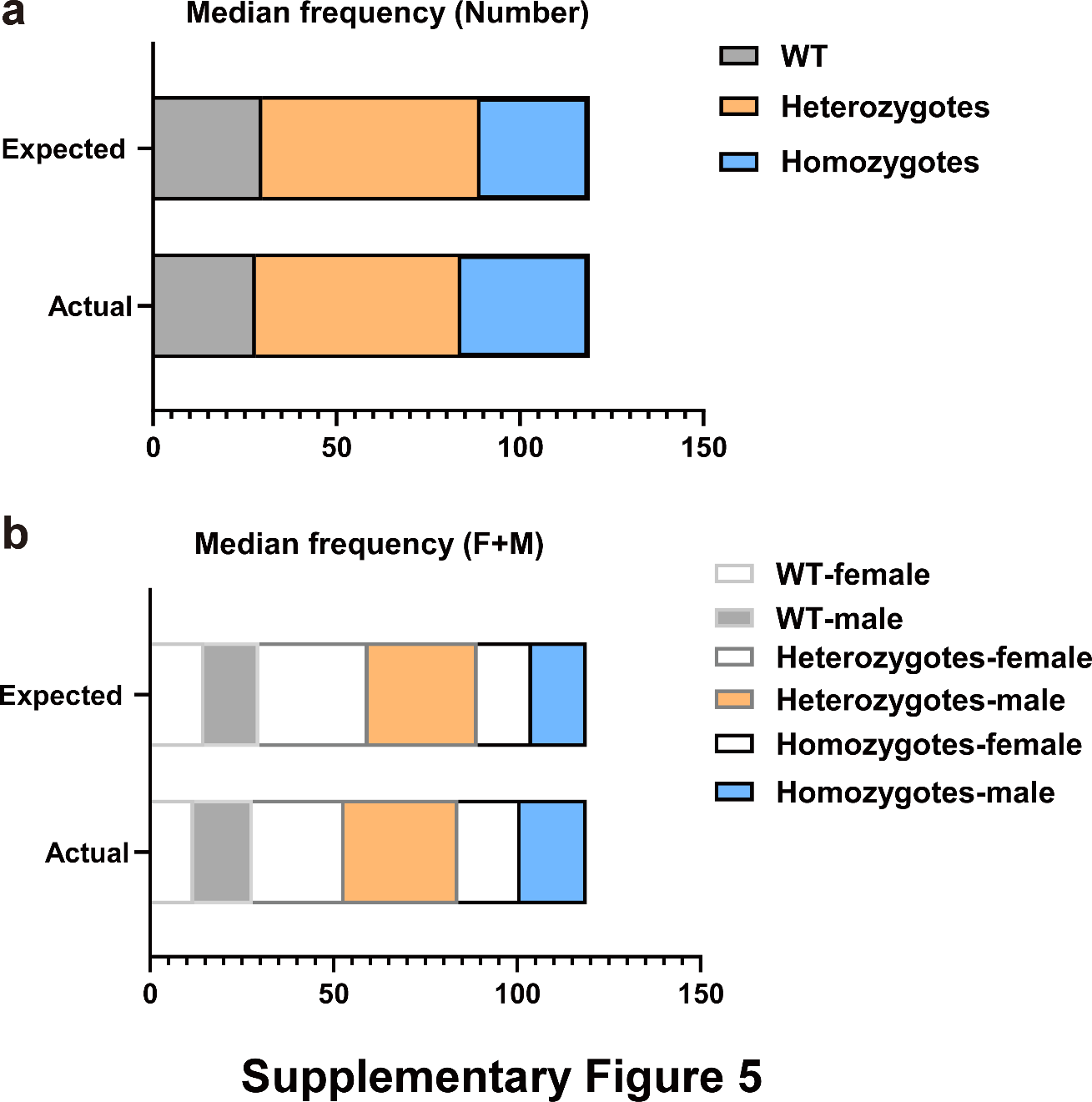
**

**Supplementary Figure 5. Median frequency of the mutant *Cntnap2*^-^*^I1254T^* knockin mice. a** *Cntnap2*^-^*^I1254T^* mice were generated by heterozygotes breeding, and the pups exhibited Mendelian frequencies of inheritance. **b** All mice showed Mendelian frequencies of inheritance, and there is no gender difference of the pups. Numbers of different mice are summarized in Supplementary Table 2.


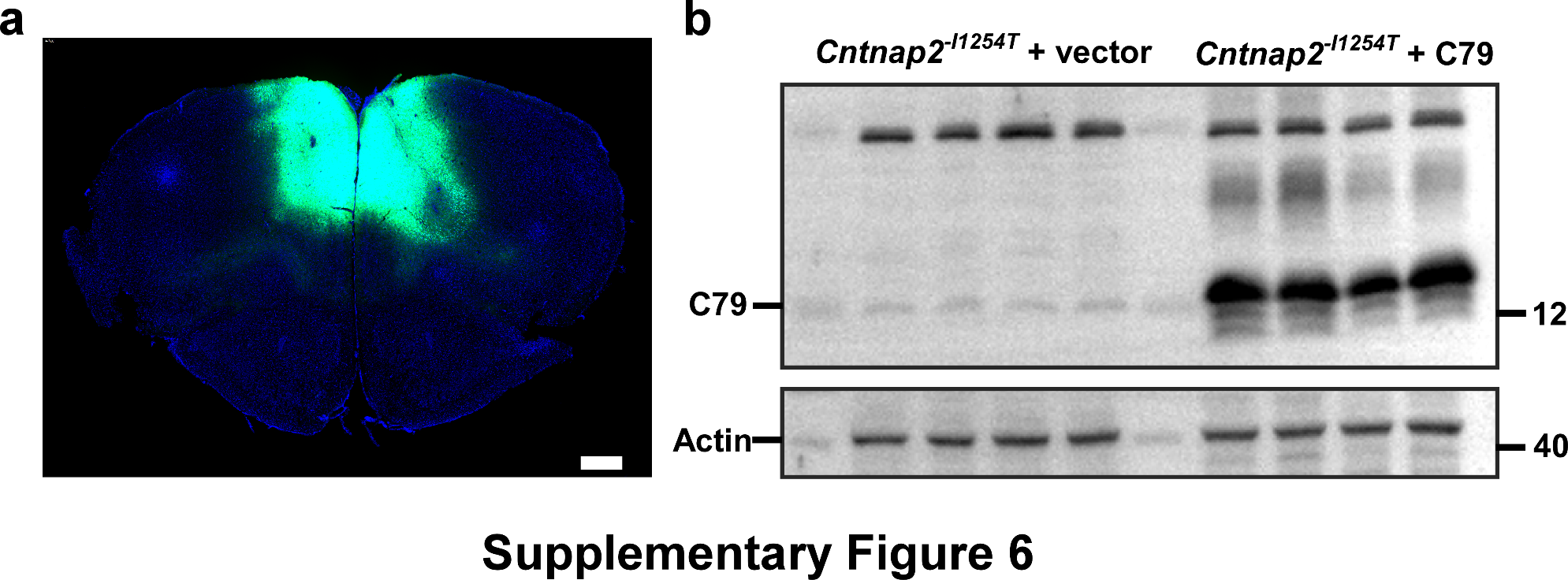


**Supplementary Figure 6. The detection of C79 in the medial prefrontal cortex (mPFC) of the mutant *Cntnap2*^-^*^I1254T^* knockin mice.** C79 protein level is detected by microscope **(a)** and Western blot **(b)**, which is significantly increased in mPFC of mutant mice injected with adeno-associated virus (AAV) vector expressing CNTNAP2-C79 protein. Scale bar = 500 μm.


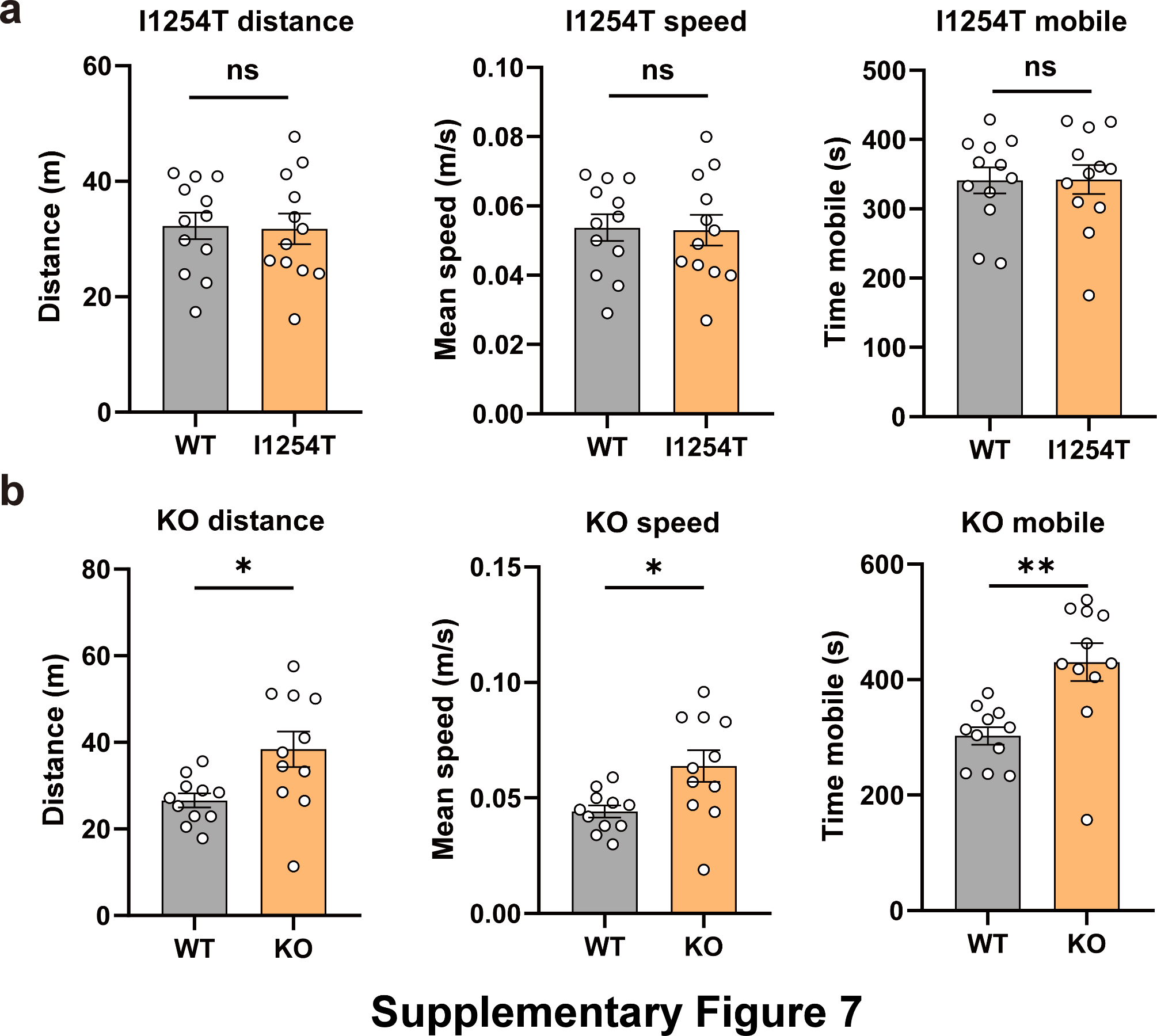


**Supplementary Figure 7. Open field test in I1254T mice and KO mice. a** *Cntnap2*^-I1254T^ mice displayed a similar level of voluntary movement to WT mice (n = 12 per group: male = 6, female = 6). **b** *Cntnap2*^-/-^ mice showed a hyper locomotor activity when compared to WT littermates (n = 11 per group: male = 6, female = 5). Statistical significance was assessed by unpaired t-test. *p < 0.05; **p < 0.01. All the results are expressed as mean ± SEM.


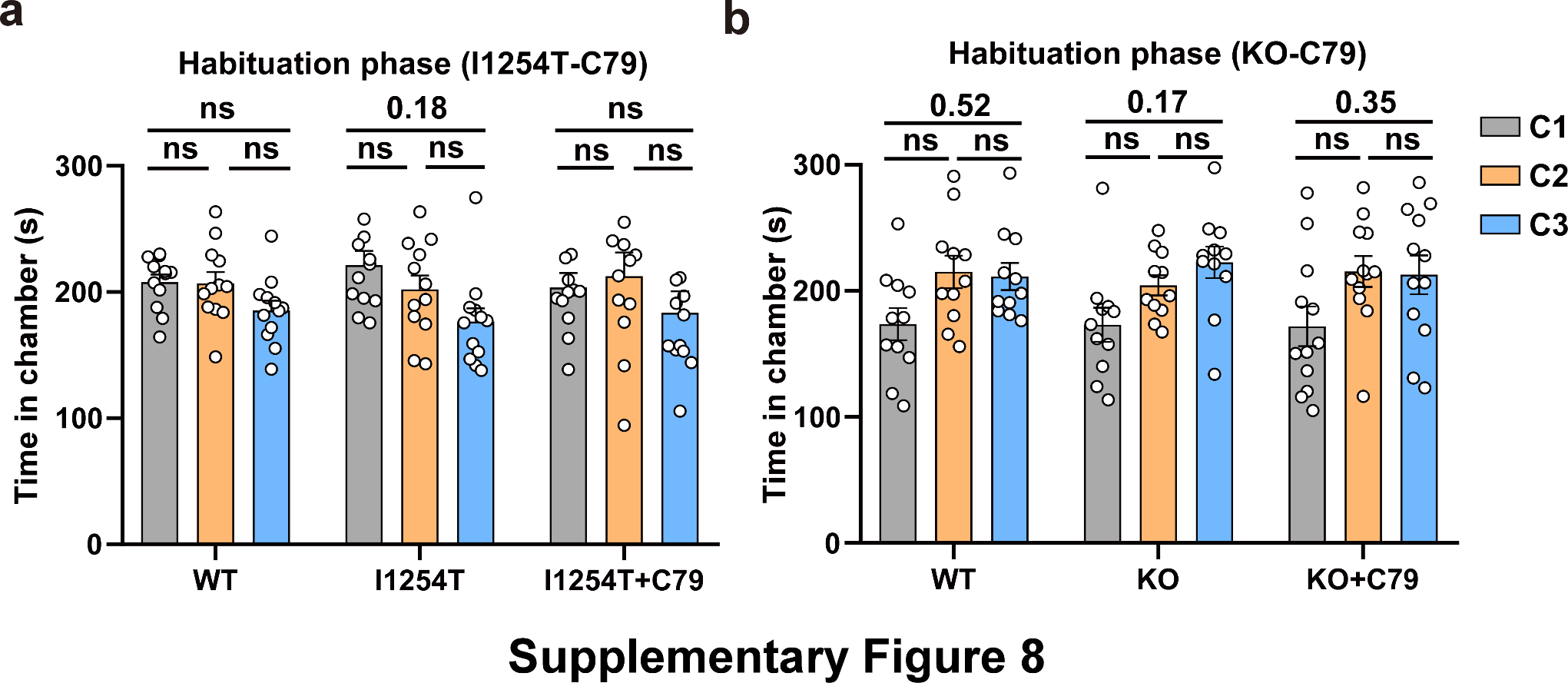


**Supplementary Figure 8. The habituation phase of three chamber sociability tests.** All mice were placed to explore three divided chambers for 10 minutes and there was no significant social preference between WT and *Cntnap2*^-I1254T^ **(a)** or *Cntnap2*^-/-^ mice **(b)** spending in each chamber .Statistical significance was assessed by two-way ANOVA followed by Bonferroni’s test. *p < 0.05. All the results are expressed as mean ± SEM.

**Supplementary Tables**

**Supplementary Table 1. Summary of the effects of each mutation**

| **Mutation** | **Impact on C79** | **Impact on C96** | **Other impact** |
| --- | --- | --- | --- |
| L99P | ns | Increased C96 by ADAM10 | ns |
| D98R | ns | Increased C96 by ADAM10 | Altered migration rate |
| LD99/98PR | ns | ns | Altered migration rate |
| H97E | ns | Reduced C96 | ns |
| DH98/97RE | Reduced C79 | Reduced C96 | Altered migration rate |
| L96P | ns | Reduced C96 | ns |
| HL97/96EP | ns | Reduced C96 | ns |
| A80P | Reduced C79 by ADAM10 | ns | ns |
| I79P | Reduced C79 | ns | ns |
| AI80/79PP | Reduced C79 | ns | ns |
| I79T | Reduced C79 | ns | ns |
| D98R/L96P/I79P | Reduced C79 | ns | Altered migration rate |
| L96P/I79P | Reduced C79 | Reduced C96 | ns |

**Supplementary Table 2. I1254T mice Mendelian ratios**

|  | **Homozygotes** | **Heterozygotes** | **WT** | **Total** |
| --- | --- | --- | --- | --- |
| **Actual** | 35 | 56 | 28 | 119 |
| **Expected** | 29.75 | 59.5 | 29.75 | 119 |
|  | **Female** | | | |
| **Actual** | 17 | 25 | 12 | 54 |
| **Expected** | 14.875 | 29.75 | 14.875 | 59.5 |
|  | **Male** | | | |
| **Actual** | 18 | 31 | 16 | 65 |
| **Expected** | 14.875 | 29.75 | 14.875 | 59.5 |

**Supplementary Table 3. Primers for site-directed mutagenesis**

| **Mutation** | **Forward Primer 5’- 3’** | **Reverse Primer 5’- 3’** |
| --- | --- | --- |
| L99P | tggcacccggatcacctggattca | tgaatccaggtgatccgggtgcca |
| D98R | tggcacctgcgtcacctggattca | tgaatccaggtgacgcaggtgcca |
| LD9998PR | tggcacccgcgtcacctggattca | tgaatccaggtgacgcgggtgcca |
| H97E | tggcacctggatgagctggattca | tgaatccagctcatccaggtgcca |
| DH9897RE | tggcacctgcgtgagctggattca | tgaatccagctcacgcaggtgcca |
| L96P | tggcacctggatcacccggattca | tgaatccgggtgatccaggtgcca |
| HL9796EP | tggcacctggatgagccggattca | tgaatccggctcatccaggtgcca |
| A80P | caaggccaacctataagaaatgga | tccatttcttataggttggccttg |
| I79P | caaggccaagctccaagaaatgga | tccatttcttggagcttggccttg |
| AI8079PP | caaggccaacctccaagaaatgga | tccatttcttggaggttggccttg |
| I79T | caaggccaagctacaagaaatgga | tccatttcttgtagcttggccttg |
| D98RL96P | tggcacctgcgtcacccggattca | tgaatccgggtgacgcaggtgcca |

Primers for PCR-based site-directed mutagenesis in generating mutant constructs containing the last 108 aa. Plasmid sequences containing mutant nucleotides (marked in red) were confirmed by Sanger Sequencing.
